# Discovery and Validation of Novel Umami Peptides from Traditional Broad Bean Paste (Doubanjiang)

**DOI:** 10.3390/foods15101819

**Published:** 2026-05-21

**Authors:** Dandan Song, Yashuai Wu, Yanfei Feng, Liang Yang

**Affiliations:** 1 School of Brewing Engineering, Moutai Institute, Renhuai 564501, China; songdd0330@163.com; 2 Guizhou Key Laboratory of Microbial Resources Exploration in Fermentation Industry, Kweichow Moutai Group, Zunyi 564501, China; 3 Modern Baijiu Brewing Technology Engineering Research Center of Guizhou Universities, Moutai Institute, Renhuai 564501, China; 4 Guizhou Province Technology Innovation Center for Jiangxiangxing Baijiu, Moutai Institute, Renhuai 564501, China; 5 School of Food Science and Engineering, South China University of Technology, Guangzhou 510640, China; wyss995418706@163.com; 6 School of Resources and Environment, Moutai Institute, Renhuai 564501, China; qianxiafyf@163.com

**Keywords:** traditional doubanjiang, umami peptides, T1R1/T1R3 receptor, molecular docking, salt reduction and umami enhancement

## Abstract

Traditional doubanjiang was investigated to identify endogenous peptides that may contribute to taste maintenance under salt-reduction conditions. Peptidomics identified 1230 peptides at −10logP ≥ 15. UMPred-FRL predicted 161 potential umami peptides, and molecular docking showed that 141 of these peptides could enter the binding site of the T1R1/T1R3 receptor. The successfully docked sequences were mainly short oligopeptides containing three to five amino acid residues. Based on docking scores, six representative candidate peptides were screened, namely EESP, SCPH, SSSGF, PDTE, SYH, and DYDS. Docking and MM-GBSA analyses suggested that these peptides mainly bound within the VFT cavity of T1R1/T1R3, and the interacting residues were dominated by polar residues such as Ser, Asn, Gln, and His and hydrophobic residues such as Tyr, Ile, Leu, and Val. MM-GBSA further suggested that vdW was the major favorable contributor, while Lipo supported complex stability. The umami thresholds of the six peptides ranged from 0.14 to 1.09 mmol/L. Experimental validation by threshold determination and sensory addition showed that all six peptides significantly increased saltiness, whereas their effects on umami differed. PDTE showed the strongest umami-enhancing effect, while SSSGF, SYH, and SCPH exhibited more pronounced saltiness synergy. These results suggest that the screened peptides do not necessarily amplify umami in complex food systems, but may contribute to taste maintenance under salt-reduction conditions through umami support, saltiness synergy, and taste-structure remodeling.

## 1. Introduction

Traditional doubanjiang is one of the most representative legume-based fermented condiments in China [1]. Compared with general broad bean paste, doubanjiang is distinguished by its chili–broad bean semi-solid fermentation system, high salt environment, long maturation period, and characteristic spicy, salty, umami-rich flavor profile. Pixian doubanjiang is a typical representative, in which fermented broad beans and salted chili are separately prepared and then mixed for further aging, giving the product a more complex aroma and taste than ordinary broad bean paste [2]. It has both regional dietary cultural significance and industrial application value. The predominant nutritional and taste-related components of doubanjiang include proteins and their hydrolysis products, carbohydrates, lipids, free amino acids, peptides, organic acids, reducing sugars, phenolic acids, flavonoids, and aroma-active compounds [2,3]. Using broad beans and other legumes as raw materials, it undergoes open, multi-microbial, and long-term fermentation [4,5]. In traditional production, the broad bean koji or meju stage is commonly associated with mold-driven proteolysis, especially Aspergillus-related fermentation, whereas the subsequent high-salt aging process is mainly an open mixed fermentation involving indigenous halotolerant bacteria and yeasts, such as Tetragenococcus, Bacillus, Zygosaccharomyces, and Candida species [3,6]. Therefore, doubanjiang fermentation is not a strictly spontaneous or strictly pure-culture process in all cases, but is better described as a starter-assisted and environment-driven multi-microbial fermentation system. During this process, proteins, starch, and lipids are continuously degraded and transformed, gradually forming amino acids, peptides, and other compounds [7]. Studies have shown that the flavor formation of doubanjiang is not the result of the accumulation of a single component. Rather, it is shaped by the coordinated effects of raw material characteristics, production process, microbial community composition and function, and metabolic networks [8,9]. During this process, taste-active compounds formed at different fermentation stages and their interactions further determine the construction and expression of the taste profile of doubanjiang. In this complex flavor system, umami is one of the important dimensions of the sensory quality of doubanjiang [10,11,12]. It is not only an important basis for sensory recognition, but also a key reflection of its flavor-supporting capacity as a compound seasoning base [13]. In addition to its sensory value, doubanjiang may also provide bioactive compounds formed during fermentation, such as antioxidant peptides, ACE-inhibitory peptides, phenolic acids, and flavonoids. These components suggest potential health-related benefits, including antioxidant and antihypertensive activities, although its high salt content means that such benefits should be considered together with sodium intake control [14,15].

Against the background of a continuing health-oriented consumption trend, traditional doubanjiang faces an increasingly acute and urgent practical challenge, namely the contradiction between high salt dependence and the demand for salt reduction. The World Health Organization recommends that daily sodium intake for adults should be below 2000 mg, which is approximately equivalent to 5 g of salt, whereas the global average sodium intake remains well above this recommended level [16]. Salt is widely present in processed foods because it is added during manufacturing not only to adjust taste, but also to regulate water activity, texture, microbial stability, enzyme activity, and shelf life. This is particularly important in fermented foods, where salt directly affects both product safety and flavor formation. In traditional fermented food systems, salt not only imparts saltiness but also plays important roles in osmotic pressure regulation, suppression of spoilage microorganisms, maintenance of enzyme activity, and control of the fermentation process [17,18]. Therefore, salt reduction in doubanjiang must balance two requirements: sufficient salt is needed to preserve the specific fermentation ecology and product safety, whereas excessive sodium intake may be harmful to human health. Partial replacement of NaCl with salt substitutes, especially KCl-containing mixed salts, has been explored as a feasible strategy for sodium reduction in Pixian douban and other fermented foods. However, the substitution ratio must be carefully controlled because excessive replacement may affect sensory quality, microbial succession, and metabolite formation [19,20].

Therefore, salt reduction is essentially not a simple decrease in NaCl addition, but a systematic intervention in the fermentation microecology, metabolic network, and flavor formation process. Several salt-reduction strategies have been tested in doubanjiang or Pixian douban systems, including direct low-salt fermentation by lowering NaCl concentration, partial replacement of NaCl with salt substitutes such as KCl-based mixed salts, and the use of selected starter cultures to improve fermentation quality and safety [21]. Existing studies have shown that salt reduction may lead to insufficient flavor, deterioration of texture, increased microbial safety risks, and the accumulation of harmful metabolites. Although direct low-salt fermentation can promote microbial metabolism and increase some flavor-related compounds, it may also disturb microbial succession and increase total acids, biogenic amines, or conditional pathogens [22]. Salt substitutes can reduce sodium intake and partly improve sensory quality, but their effects depend strongly on the substitution ratio and may introduce bitterness, metallic notes, or changes in fermentation characteristics. Starter-culture regulation can improve microbial safety and flavor formation to some extent, but it does not fully explain how endogenous taste-active molecules compensate for salt reduction. Therefore, existing strategies remain insufficient for achieving simultaneous sodium reduction, flavor maintenance, and safety control, and a molecular-level understanding of endogenous umami or saltiness-enhancing factors is still needed [8,23,24]. In douban-type fermentation systems in particular, excessively low salinity may accelerate metabolism, but may also promote the accumulation of biogenic amines and introduce potential safety risks [25,26]. This suggests that the establishment of salt-reduction strategies for doubanjiang should simultaneously address the coordinated optimization of flavor compensation and safety control [27].

Against this background, umami peptides have attracted increasing attention. Compared with traditional single-taste compounds, umami peptides are naturally derived and structurally diverse [28]. They can directly contribute to umami and may also exhibit umami-enhancing or saltiness-enhancing effects [29]. Therefore, they are of great significance for salt reduction and umami enhancement [3,30]. Relevant studies have indicated that umami peptides are widely present in fermented foods, and their taste perception is closely associated with recognition by the T1R1/T1R3 receptor [31,32]. In the doubanjiang system, experimental evidence has shown that multiple novel peptides with umami, umami-enhancing, and saltiness-enhancing functions can be isolated from fermented broad bean paste [12]. Subsequent studies combining peptidomics, machine learning, and molecular docking further screened multiple low-threshold umami peptides from soybean paste and Pixian doubanjiang. Compared with other fermented condiments, doubanjiang appears to share a common fermentation-driven peptide basis for umami formation, but it also shows matrix-specific features. In soy sauce, sensory-guided separation and LC-MS/MS studies have identified small umami peptides, including peptides below 1 kDa with low taste thresholds, indicating that short peptides can directly contribute to the strong umami profile of liquid fermented condiments. In miso, umami intensity is more often associated with proteolysis-derived amino acids, especially glutamate and aspartate, together with diverse fermentation products, resulting in a multidimensional umami profile rather than a peptide-only contribution. By contrast, doubanjiang and Pixian doubanjiang are semi-solid, chili–broad bean fermentation systems. Their peptide profiles are affected not only by legume protein hydrolysis but also by high salt, long aging, and microbial metabolism. This may explain why peptides in doubanjiang may contribute not only to umami intensity, but also to saltiness enhancement and overall taste-body construction. These findings indicate that traditional doubanjiang indeed contains abundant endogenous umami resources that have not yet been fully utilized [12,33].

On this basis, the present study focused on traditional doubanjiang and addressed the core issue of umami retention and quality improvement under salt-reduction conditions. Although previous studies have shown that microbial succession, enzymatic hydrolysis, and metabolite accumulation jointly drive the formation of taste-active compounds in doubanjiang and other fermented bean condiments, the endogenous peptide basis responsible for umami maintenance and saltiness compensation remains insufficiently clarified. The emphasis was not placed on the simple introduction of exogenous flavor enhancers. Instead, attention was directed toward the product itself to re-identify and mine endogenous peptide molecules generated during fermentation that can support the umami framework. Therefore, the research gap lies in the lack of an integrated understanding of how fermentation-derived peptides are identified, predicted, receptor-recognized, and experimentally validated in a real doubanjiang matrix under salt-reduction-oriented flavor regulation. The objective of this study was to establish a peptide-centered evidence chain by integrating peptidomics, umami prediction, T1R1/T1R3 docking, MM-GBSA analysis, threshold determination, and sensory addition validation. Through this approach, representative endogenous umami peptides were screened, and their potential roles in umami support, saltiness synergy, and taste-structure remodeling were clarified. This study is expected to provide a more targeted theoretical basis for salt-reduction upgrading and quality optimization of traditional doubanjiang. It may also offer new theoretical support and practical ideas for the development of natural flavor enhancers and for shifting salt-reduction strategies for traditional fermented condiments from empirical adjustment to molecularly guided precision design.

## 2. Materials and Methods

### 2.1. Samples and Reagents

A traditional doubanjiang sample was used in this study. The product was a Pixian-type doubanjiang paste produced in Pidu District, Chengdu, Sichuan Province, China, which is the typical geographical origin of Pixian doubanjiang. According to the supplier information, the broad bean kernels used for production originated from the main broad bean-producing areas used for Pixian doubanjiang manufacture, such as Sichuan or Yunnan Province. Its ingredients were red pepper, broad bean kernels, edible salt, water, wheat flour, and rapeseed oil. The typical Pixian doubanjiang process includes the preparation of broad bean meju, salted chili fermentation, and mixed ripening fermentation. Briefly, broad beans are soaked, steamed, mixed with wheat flour, inoculated with Aspergillus oryzae, and fermented with 10–12% salt for about 2–3 months to prepare meju. Red pepper is cut into small pieces of approximately 1–2 cm and fermented with 10–15% salt. The meju is then mixed with red pepper and brine containing about 20% salt, followed by open ripening fermentation for several months or years. Mature products are commonly obtained after 12–18 months, whereas deep-mature products are usually fermented for more than 24 months. After collection, the sample was immediately stored at 0 °C under refrigeration. To reduce within-batch heterogeneity, samples were taken from the upper, middle, and lower positions of the container after thorough mixing, combined into a composite sample, and then divided for subsequent analysis. Three technical replicates were prepared from the composite sample for peptide extraction and LC-MS/MS analysis. Because the present study aimed to identify endogenous umami peptides in a representative mature doubanjiang sample, batch-to-batch variability was not used as the main experimental factor. This limitation has been clarified, and further studies should include multiple independent production batches to verify the stability of the identified peptides across different raw material origins and fermentation conditions. Sampling time was recorded using 24 h as 1 d.

The main reagents used in the experiment included LC/MS-grade water, LC/MS-grade acetonitrile, and LC/MS-grade formic acid, all purchased from Thermo Fisher Scientific (Waltham, MA, USA). Methanol and chloroform were purchased from Sinopharm Chemical Reagent Co., Ltd. (Shanghai, China). Pierce™ trifluoroacetic acid was purchased from Thermo Fisher Scientific. The desalting material was a 3M disk-C18 (3M Bioanalytical Technologies, St. Paul, MN, USA). Ultrafiltration was performed using Millipore ultrafiltration centrifuge tubes (MilliporeSigma, Burlington, MA, USA) with molecular weight cutoffs of 10 and 3 kDa. The umami peptides used were EESP, SCPH, SSSGF, PDTE, SYH, and DYDS, with purities of at least 98%, and were purchased from Gil Biochemical Co., Ltd. (Shanghai, China).

### 2.2. Instruments

The instruments used for sample pretreatment included an ICES-70 ice maker from Xiamen Guoyi Scientific Instrument Co., Ltd. (Xiamen, China), a Vortex-Genie 2 vortex mixer from Scientific Industries, Inc. (Bohemia, NY, USA), a DW-86W150 medical low-temperature storage refrigerator from Qingdao Aucma Global Medical Co., Ltd. (Qingdao, China), an ABS-MS-078 constant-temperature mixer from Hefei Aibensen Scientific Instrument Co., Ltd. (Hefei, China), a Research plus manual single-channel pipette from Eppendorf (Hamburg, Germany), a Centrifuge 5430R refrigerated centrifuge from Eppendorf (Hamburg, Germany), an LNG-T98 refrigerated centrifugal vacuum concentrator and an LNG-T88 benchtop rapid centrifugal vacuum concentrator, both from Taicang Huamei Biochemical Instrument Factory (Taicang, China), a Fielda-650D ultrasonic cell disruptor from Jiangsu Bochang Intelligent Technology Co., Ltd. (Wuxi, China), an MS105DU analytical balance from METTLER TOLEDO (Greifensee, Switzerland), and a wonbio-96D high-capacity cryogenic grinder from Shanghai Wonbio Biotechnology Co., Ltd. (Shanghai, China). Peptide quantification was performed using a Thermo Scientific NanoDrop One microvolume UV spectrophotometer (Thermo Fisher Scientific, Waltham, MA, USA). Liquid-phase separation was carried out on a Thermo Scientific Vanquish Neo UHPLC system (Thermo Fisher Scientific, Waltham, MA, USA). Mass spectrometric detection was performed using a Thermo Scientific Orbitrap Astral mass spectrometer (Thermo Fisher Scientific, Waltham, MA, USA).

### 2.3. Experimental Methods

#### 2.3.1. Pretreatment

An appropriate amount of doubanjiang sample was taken for peptide extraction. Methanol, chloroform, and water were added sequentially at a volume ratio of 3:1:4. After extraction, the extract was dried and then redissolved. The sample was then preliminarily quantified using a NanoDrop One microvolume UV spectrophotometer. Considering that UV-based peptide quantification may be affected by peptide sequence composition, aromatic residue content, residual salts, and solvent background, the NanoDrop result was not used as an absolute measure of peptide abundance. It was used only for pre-loading normalization. To improve loading consistency, all peptide extracts were processed under the same extraction, desalting, drying, and reconstitution conditions. The final peptide solutions were prepared in 0.1% formic acid, and the same solvent was used as the blank. Each sample was measured repeatedly, and the injection volume was adjusted according to the measured peptide concentration so that an equal calculated peptide amount was loaded for LC-MS/MS analysis. In addition, chromatographic signal stability and total ion current profiles were checked after data acquisition to exclude obvious loading deviation. This strategy was adopted because previous MS-based proteomics studies have shown that equal peptide loading before LC-MS/MS can reduce technical variation and improve reproducibility, whereas UV microvolume measurement alone should be treated as a rapid quality-control and normalization tool rather than a definitive absolute quantification method [34,35].

Equal amounts of samples were subjected to desalting with 3M C18 material. Before desalting, the column was activated twice with 50 μL acetonitrile. It was then equilibrated once with 50 μL of 40% acetonitrile containing 0.1% TFA and twice with 50 μL of 2% acetonitrile containing 0.1% TFA. Before loading, the sample pH was adjusted to below 3. The sample was then loaded twice. After loading, the column was washed 2 to 3 times with 50 to 100 μL of 2% acetonitrile containing 0.1% TFA. Elution was finally performed once with 50 μL of 40% acetonitrile containing 0.1% TFA. The eluate was collected and dried. The dried peptide sample was fully dissolved in 0.1% aqueous formic acid. Peptide quantification was then performed again using NanoDrop One for subsequent LC-MS/MS analysis.

#### 2.3.2. RPLC-MS Analytical Conditions

Equal amounts of peptide samples were dissolved in mass spectrometry loading buffer and then subjected to analysis. This RPLC-MS method was designed for untargeted qualitative peptidomics rather than absolute quantification of each endogenous peptide. Therefore, external authentic standards for all identified peptides were not used, because most endogenous doubanjiang peptides were unknown before MS identification and corresponding purified standards were not commercially available. To monitor analytical stability, external system-suitability control was conducted before sample acquisition using a peptide standard mixture, and blank injections were inserted to check carryover. The system was accepted only when the standard injection showed stable retention behavior, normal peak shape, and no obvious signal loss. In addition, mass accuracy, total ion chromatogram stability, and repeated detection of high-abundance peptide signals were checked during data acquisition. This strategy is consistent with LC-MS-based peptidomics and proteomics studies, in which external standards are commonly used for system suitability and retention-time performance, whereas authentic peptide standards or isotope-labeled standards are mainly required for targeted absolute quantification [36,37]. Liquid-phase separation was performed on a Thermo Scientific Vanquish Neo UHPLC system. Data were acquired using Thermo Xcalibur 4.7. The analytical column was a homemade column with dimensions of 15 cm × 100 μm and a particle size of 1.7 μm. Mobile phase A was 2% acetonitrile in water containing 0.1% formic acid. Mobile phase B was 80% acetonitrile in water containing 0.1% formic acid. The gradient program was set as follows. At 0 min, mobile phase B was 8% and the flow rate was 1 μL/min. At 1 min, mobile phase B was increased to 17% and the flow rate remained at 1 μL/min. At 5.5 min, mobile phase B was increased to 55% and the flow rate was adjusted to 0.4 μL/min. At 7 min, mobile phase B was increased to 99% and the flow rate was adjusted to 0.7 μL/min. At 8 min, mobile phase B was maintained at 99% and the flow rate was 1 μL/min. Mass spectrometric detection was carried out using a Thermo Scientific Orbitrap Astral mass spectrometer in DDA mode. The MS1 scan range was 380 to 980 *m*/*z*. The MS2 scan range was 150 to 2000 *m*/*z*. A Top 100 method was used to select the 100 most intense precursor ions for fragmentation. The MS1 resolution was set to 240,000. The AGC target was 500%, and the maximum injection time was 3 ms. HCD was used for fragmentation. The MS2 resolution was set to 80,000 to 100,000. The AGC target was set to standard. The maximum injection time was 10 ms, and the dynamic exclusion time was 12 s. Raw mass spectrometry data files were saved in .raw format.

#### 2.3.3. Qualitative Identification of Peptides in Doubanjiang

Qualitative identification of peptides in the doubanjiang sample and analysis of their precursor protein sources were completed on the PEAKS Studio platform [38]. Based on the ingredient composition of the sample and the characteristics of raw materials used in traditional doubanjiang fermentation, an integrated reference database strategy was adopted. UniProt reference proteome FASTA sequences of broad bean Vicia faba, wheat Triticum aestivum, and red pepper Capsicum annuum were included for combined database searching. Because rapeseed oil mainly provides lipids in this system and its protein contribution is relatively limited, it was not included as a major protein source in database construction. Doubanjiang is a high-salt and long-term complex fermentation system. During fermentation, raw material proteins are continuously hydrolyzed by endogenous enzymes and microbial proteases. Peptide cleavage is therefore usually irregular. Accordingly, the digestion mode was set to Enzyme None, and matching was performed under a nonspecific digestion setting. To improve the identification of low-abundance peptides, short peptides, and potential novel sequences, the de novo module was simultaneously enabled, and de novo tag information was incorporated into the subsequent database matching process. The mass accuracy thresholds were set to 10 ppm for precursor ions and 0.03 Da for fragment ions to match the high-resolution mass spectrometry data used in this study. Variable modifications included methionine oxidation, protein N-terminal acetylation, N-terminal glutamine or glutamic acid pyroglutamylation, asparagine or glutamine deamidation, and cysteine modifications related to sample processing. A target-decoy strategy was used to control false positives. Results were filtered at 1% FDR at both the peptide-spectrum match and protein levels to ensure the reliability and accuracy of peptide identification [39].

#### 2.3.4. Efficient Screening of Potential Umami Peptides Using Machine Learning

After qualitative identification of peptides in the doubanjiang sample, the obtained peptide sequences were organized in FASTA format (Supplementary S1) and imported into the UMPred-FRL [40] online server for screening of potential umami peptides. This model is a sequence-based machine learning meta-predictor. It can rapidly determine whether a peptide has umami potential without relying on the three-dimensional structure of the protein. It is suitable for large-scale preliminary screening of candidate umami peptides in complex food systems. In this study, peptides with a UMPred-FRL output Probability greater than 0.9 were defined as candidates with high umami potential and were retained for subsequent analysis. This cutoff was selected to obtain a high-confidence candidate set and to reduce the inclusion of weakly predicted sequences during downstream molecular docking and sensory validation. The original UMPred-FRL study reported that the model achieved good performance on an independent test set, with ACC, BACC, Sn, Sp, MCC, and AUC values of 0.888, 0.860, 0.786, 0.934, 0.735, and 0.919, respectively, and was designed as a cost-effective high-throughput tool for identifying peptides with potential umami sensory properties [40].

Its core strategy is based on feature representation learning. Seven types of sequence feature encodings were combined with six commonly used machine learning algorithms to construct 42 baseline models. On this basis, new probabilistic features were generated, and a final SVM meta-predictor was established to improve the recognition of umami peptides. Studies have reported that the model achieved a balanced accuracy of 0.860, a sensitivity of 0.786, and a Matthews correlation coefficient of 0.735 on an independent test set. Its overall predictive performance was superior to that of existing methods. Therefore, this platform was used in the present study as a high-throughput screening tool for peptides derived from doubanjiang. Candidate peptides with high umami potential were retained according to the server output and were subjected to subsequent sensory evaluation, molecular docking, and functional validation to improve the specificity and efficiency of potential umami peptide discovery.

As shown in Supplementary Table S1, 45 experimentally reported umami or umami-enhancing peptides from fermented foods were collected as an independent positive-control dataset. These peptides covered different fermented matrices, including soy sauce, fermented broad bean paste, douchi, sufu, tauco, Huangjiu, fermented grains, and fermented fish products. After evaluation by UMPred-FRL, all 45 peptides were classified as umami peptides [41,42,43,44,45,46,47,48,49,50,51,52]. This result indicates that the model showed good recognition ability for known fermented-food-derived umami peptides. It also supports the reliability of UMPred-FRL as a high-throughput prescreening tool in the present study. However, model prediction was not treated as direct functional confirmation. The candidate peptides were further evaluated by receptor docking, MM-GBSA analysis, threshold determination, and sensory addition tests to ensure that the final interpretation was supported by both computational and experimental evidence.

#### 2.3.5. Molecular Docking

A homology modeling strategy was used to construct the three-dimensional structure model of the umami-related receptor. The reference sequences of T1R1/T1R3 were obtained from UniProtKB for modeling [39]. Because mGluR1 belongs to class C G protein-coupled receptors and its extracellular Venus flytrap domain directly participates in ligand recognition, and because 1EWK in the RCSB PDB has resolved the crystal structure of the extracellular ligand-binding region of this receptor in complex with glutamate, 1EWK was selected as the template on a clear structural basis. The key point of this step was not only sequence import and template calling, but also the careful treatment of conserved sites, insertion and deletion regions, and the correspondence of pocket-adjacent residues during alignment, so that the propagated influence of local mismatches on the geometry of the binding cavity could be minimized as much as possible. SWISS-MODEL was used with a template-dependent automated homology-modeling workflow. Based on sequence alignment, it can complete main-chain construction, insertion and deletion modeling, side-chain reconstruction, and energy optimization. It was therefore suitable for receptor structure prediction supported by an available experimental template. After model generation, stereochemical quality was evaluated using the SAVES v6.1 platform. The distribution of backbone φ and ψ dihedral angles in favored, allowed, and disallowed regions was assessed mainly using the Ramachandran plot. Attention was also paid to whether unreasonable conformations appeared in key residues around the binding pocket. This served as structural quality control before molecular docking [12,53].

After the receptor model met the basic quality requirements, subsequent molecular docking was carried out on the Maestro 14.5 platform. The receptor was first standardized using Protein Preparation Wizard. This included hydrogen addition, correction of bond orders and valence states, unification of residue forms, optimization of the hydrogen-bond network, and restrained minimization under the OPLS4 force field to relieve local geometric conflicts that may have remained after modeling or import. Solvent molecules and heterologous components that were not directly associated with the recognition site in the crystal environment were removed. Structural components that might contribute to local stability were retained after judgment based on pocket position and interaction context. Candidate peptides were then standardized using LigPrep in combination with Epik. Reasonable protonation states, tautomers, and low-energy conformations were generated at pH 7.0 ± 2.0 to ensure comparability of docking inputs in charge distribution, ionization state, and stereochemical configuration. A docking grid was then generated with the natural ligand-binding region of the receptor as the center. It covered the main binding cavity and the adjacent recognition space to avoid omission of possible polar anchoring regions or hydrophobic auxiliary recognition regions caused by an overly narrow grid range. Docking was then performed using the Glide workflow. The first round of conformational sampling and scoring was completed in SP mode. Post-docking minimization was carried out for the retained favorable binding poses. Representative binding conformations were finally screened according to docking scores, interaction types with key residues, and clustering results based on heavy-atom root mean square deviation. The receptor–ligand recognition pattern was then visualized and discussed mechanistically in combination with hydrogen bonds, salt bridges, cation–π and aromatic interactions, as well as hydrophobic and van der Waals contacts.

To validate the reliability of the docking protocol, reference docking was performed using known umami ligands before docking the candidate peptides. L-glutamate, MSG, IMP, and GMP were selected because amino acids and 5′-ribonucleotides are well-established natural ligands or synergistic ligands of the T1R1/T1R3 umami receptor [54]. The same receptor model, grid region, ligand preparation procedure, and Glide parameters were used for these reference ligands and for the candidate peptides. The validation results show that the known ligands could enter the VFT recognition cavity and form stable polar interactions with residues reported to participate in umami ligand recognition. This supported the rationality of the binding pocket definition and the docking parameter setting. The docking scores were therefore used for relative comparison and candidate prioritization, rather than as direct evidence of sensory activity. Final functional interpretation was further based on MM-GBSA analysis, threshold determination, and sensory addition validation.

#### 2.3.6. MM/GBSA Binding Free Energy Calculation

In the Maestro v14.5 environment, the Prime module was used to estimate the MM-GBSA binding free energies of T1R1/T1R3-candidate peptide complexes in order to evaluate the relative binding stability between different peptides and the receptor. This method is based on molecular mechanics and an implicit solvent model. The OPLS4 force field was used for all receptor, peptide, and complex energy calculations to maintain consistency with the receptor preparation, ligand preparation, and docking workflow. OPLS4 was selected because it has been developed and validated for improved treatment of challenging chemical space, including charged groups, heteroatom-containing moieties, and protein-ligand systems, which are relevant to short peptides containing acidic, polar, aromatic, and sulfur-containing residues. The favorable conformations obtained from molecular docking were first subjected to restrained local minimization. During minimization, residues within 5 Å of the ligand were allowed to relax, whereas the remaining receptor structure was restrained to preserve the docking-derived binding mode. The energies of the complex, receptor, and ligand were then calculated separately under a unified force field and solvent condition. The VSGB implicit solvation model implemented in Prime was applied to estimate polar solvation energy, while the nonpolar solvation contribution was calculated from the solvent-accessible surface area term. In accordance with common MM-GBSA practice, the solute dielectric constant was set to 1.0 and the solvent dielectric constant was set to 80.0 to represent an aqueous environment. These settings were chosen because MM-GBSA with a low solute dielectric is commonly used for relative binding free-energy ranking, and VSGB-type generalized Born models have been widely applied for protein-ligand endpoint rescoring. Binding free energy was calculated according to ΔGbind = Gcomplex − Greceptor − Gligand. Binding free energy is jointly determined by van der Waals interactions, electrostatic interactions, and polar and nonpolar solvation contributions. It can therefore be used to compare the affinity trends of candidate peptides toward T1R1/T1R3 from an energetic perspective. For clarity, the decomposed energy terms included Coulomb, vdW, Hbond, Lipo, Packing, and Solv GB. The entropy term was not included, so the calculated ΔGbind values were interpreted as relative endpoint scores rather than absolute experimental binding free energies. It should be noted that MM-GBSA is an endpoint energy evaluation method. Its results are more suitable for relative ranking and auxiliary screening of candidate peptides within the same system, and should not be directly regarded as rigorous experimental free energy values [39,55].

#### 2.3.7. Determination of Taste Thresholds of Umami Peptides

The taste thresholds of the target umami peptides were determined using the TDA taste gradient dilution method combined with the three-cup test. A stock solution of each umami peptide was first prepared at pH 6.5 and a mass concentration of 1 mg/mL. It was then serially diluted with deionized water at a 1:1 ratio to obtain a series of gradient samples from low to high concentration. Sensory evaluation was performed by 10 systematically trained assessors. The panel consisted of assessors with previous experience in sensory evaluation of fermented condiments or basic taste discrimination. Before formal testing, all assessors completed training in the recognition of 5 basic tastes using reference solutions of sucrose for sweetness, NaCl for saltiness, citric acid for sourness, caffeine for bitterness, and MSG for umami. The training was conducted over 3 sessions. Each session included taste recognition, intensity ranking, and repeated three-cup discrimination tests. Only assessors who could correctly identify the umami reference and distinguish the peptide-containing sample from blanks in repeated training tests were included in the formal panel. During testing, the gradient samples were presented sequentially from low to high concentration. At each gradient, a three-cup test was applied, consisting of 2 blank controls and 1 sample to be tested. The cups were coded in random order and presented to the assessors for identification [42,56,57]. The assessors were required to identify the different samples in each set of three cups and record the lowest concentration at which the target sample could first be stably recognized. To reduce the interference of positional bias, order effects, and oral residue on judgment, deionized water was used for mouth rinsing between gradients, and an appropriate interval was set before the next test group. Threshold determination for each peptide was conducted in 3 independent sensory replicates on separate testing sessions. In each replicate, all dilution gradients were freshly prepared and independently coded. A concentration was accepted as the recognition threshold only when the target sample was correctly identified by at least 6 of the 10 assessors and the result was reproduced in the repeated verification test. For the concentration gradient that initially met the identification requirement, repeated verification was conducted using the same group of samples. When one gradient could be correctly identified, whereas the preceding gradient could not be stably distinguished, this critical interval was taken as the basis for threshold determination and was used to characterize the lower limit of taste detection for the umami peptide [58,59].

#### 2.3.8. Sensory Evaluation of Doubanjiang

Accurately weigh out 2000 g of low-salt system broad bean paste (i.e., the salt usage is reduced by 40% during the production process), thoroughly mix it, then take 250 g of the mixture as the blank control sample, denoted as A. Six additional portions of 250 g each were then taken from the remaining sample as test samples for umami peptide addition. The unportioned remainder was sealed for short-term standby use. According to the predetermined order, 1 mL of each novel umami peptide solution at its corresponding umami threshold was added to the 6 test samples. The peptide sequences were EESP, SCPH, SSSGF, PDTE, SYH, and DYDS. After addition, each sample was immediately mixed thoroughly to ensure uniform dispersion of the peptide solution in the doubanjiang matrix and was then labeled A-1 to A-6. The same 10 trained assessors were used for doubanjiang sensory evaluation to maintain consistency with the threshold test. Before sample scoring, the panel received an additional product-specific training session using low-salt doubanjiang, regular doubanjiang, and peptide-added pilot samples. The training focused on the definitions of umami, saltiness, sour-umami, bitterness, bitter aftertaste, drying sensation, taste body, and aftertaste persistence. The scoring criteria in Table 1 were explained and calibrated before formal evaluation. Before sensory evaluation, all samples were randomly coded with 3-digit numbers and presented in a balanced random order. Each assessor evaluated 7 samples, including the control and 6 peptide-added samples. Each sample was evaluated in 3 independent replicates. The mean value of the 3 replicates was used for statistical analysis. To avoid carryover effects from the high-salt and strongly fermented matrix, no more than 7 samples were evaluated in one session. Drinking water was used for mouth rinsing between samples, and a fixed interval was set before the next sample. The evaluation was conducted under consistent room conditions, and assessors were not informed of sample identity or peptide addition.

Before sensory evaluation, all samples were recoded using random codes and were presented and evaluated under identical conditions. Parallel replicates were included during evaluation. Drinking water was used for mouth rinsing between samples to minimize interference from residue left by the previous sample. In sensory studies of doubanjiang and related compound seasoning systems, trained panelists, random coding, and the 9-point intensity scale are commonly used to improve the repeatability and comparability of results [7,10,17].

For doubanjiang, umami is not a single taste stimulus. It is a complex taste sensation jointly formed by free amino acids, umami peptides, and some organic acids. Recent studies have shown that a large number of free peptides accumulate during the maturation of fermented broad bean paste. Increases in umami peptides, amino acids, and organic acids are closely associated with enhanced umami perception, and some peptides also exhibit umami-enhancing effects. Therefore, in sensory evaluation, the judgment of umami should not rely only on the immediate intensity at the moment of tasting. Greater attention should be paid to taste body, oral spreading, persistence in the later stage, and coordination with sauce aroma and fermentation aroma. The umami of high-quality doubanjiang is usually characterized by a natural onset, full-bodied taste, and long-lasting aftertaste, without abrupt, thin, or harsh taste impressions [10].

Saltiness is an important part of the taste framework of doubanjiang, but a high score does not simply mean a stronger salt sensation. Existing studies have shown that the dominant tastes of doubanjiang are umami, saltiness, and sourness. Appropriate salt regulation can reduce harsh saltiness while improving umami and mellowness, indicating that saltiness evaluation should emphasize both intensity and quality. In practical evaluation, attention should focus on whether the saltiness is clear, clean, and rounded, whether it is coordinated with umami and fermented taste, and whether sharpness, roughness, bitterness, or suppression of other tastes is present. Therefore, a high saltiness score for doubanjiang is more appropriate for samples with distinct but not excessive saltiness, with supporting taste at the entrance, without lingering heaviness in the aftertaste, and with good coordination with the overall flavor, rather than for samples showing the strongest salt stimulus alone.

With reference to the 9-point intensity scale commonly used in sensory studies of doubanjiang and related foods, 0 can be set as no perception and 9 as extremely strong, with the intermediate scores increasing according to intensity. Applications of both the 0 to 9 scale and the 9-point intensity scale have been reported in doubanjiang studies. Therefore, Table 1 below was used in this study as the specific scoring basis for umami and saltiness.

## 3. Results and Analysis

### 3.1. Qualitative Identification of Peptides in Doubanjiang and Analysis of Their Potential Contribution to Umami Flavor

In this study, a confidence threshold of −10logP ≥ 15 was set for the peptides obtained by database searching, and a total of 1230 peptides were identified (Supplementary S2). Their umami properties were then predicted using UMPred-FRL with a threshold of 0.9. A total of 161 potential umami peptides were identified (Table 2).

As shown in Table 2, statistical analysis of the 1230 identified peptide sequences revealed that this peptide library exhibited pronounced low-molecular-weight characteristics. Peptide length ranged from 2 to 28 aa, with an average length of 4.81 aa and a median length of 4 aa. Among them, 659 were tetrapeptides, accounting for 53.7%. Peptides with lengths of 3 to 5 aa totaled 1090, accounting for 88.8%, and sequences within 10 aa accounted for 93.2%. This distribution pattern, dominated by short-chain peptides, is consistent with the formation of a large number of low-molecular-weight peptides through continuous protein hydrolysis in the peptidomes of fermented foods. In terms of amino acid composition, S, P, G, E, L, F, Y, D, N, and H occurred frequently. Among them, S, P, and G appeared 530, 496, and 494 times, respectively, indicating that this peptide library was enriched in flexible residues, turn-forming residues, and acidic residues. When classified by physicochemical properties, small residues A, G, S, P, and T accounted for 35.9%, hydrophobic residues A, V, L, I, M, F, W, and Y accounted for 36.4%, acidic residues D and E accounted for 12.2%, and aromatic residues F, W, and Y accounted for 14.3%. These results indicate that the identified sequences had marked structural heterogeneity and amphiphilic potential. Previous studies have shown that wheat storage proteins are generally rich in glutamine, glutamic acid, and proline, whereas broad bean proteins are rich in glutamic acid, aspartic acid, and polar residues such as Ser and Thr. This is consistent with the enrichment profile of Q, E, P, D, S, and T observed in this study. Clear terminal preferences were also observed [60,61]. The N-termini were mainly Y, M, E, D, and F, whereas the C-termini were concentrated in F, H, P, Y, R, and E. This suggests that the peptide library was not generated by random fragmentation, but was more likely shaped by initial endoprotease cleavage followed by continuous trimming by exopeptidases. Among dipeptide and tripeptide motifs, fragments such as SS, PP, GS, SG, GN, EF, NQL, GNP, GNQ, and QEQ were repeatedly observed. This further indicates that local structures related to serine, proline, glycine, and glutamine showed high retention during degradation. At the same time, multiple groups of nested homologous fragments were clearly observed, such as YLAGNQ to YLAGNQEQEFLRY, YLGGNP to YLGGNPEVEFPETQE, GNQEQEF to GNQEQEFLRY, ATPADVLANAF to ATPADVLANAFGLR, and DFLEDAL to DFLEDALNVNRHIVDR. These results reflect continuous truncation and stagewise accumulation of the precursor proteins. Peptidomics and terminome analyses have suggested that such nested fragments are important sequence evidence of progressive protein degradation.

Sequence statistics of the 161 screened potential umami peptides using Probability > 0.9 as the cutoff (Table 2) further showed that this candidate set had relatively concentrated structural features. This stringent cutoff was selected to reduce the inclusion of weakly predicted sequences during large-scale screening, because UMPred-FRL is a sequence-based meta-predictor designed for high-throughput identification rather than direct sensory confirmation. The original UMPred-FRL study reported good performance on an independent test set, with ACC, BACC, Sn, Sp, MCC, and AUC values of 0.888, 0.860, 0.786, 0.934, 0.735, and 0.919, respectively, supporting its use as a preliminary screening tool. A probability threshold of 0.9 was therefore used to prioritize peptides with high prediction confidence and to control the downstream workload of docking and sensory validation. Peptide length ranged from 3 to 21 aa, with an average length of 5.13 aa. Short-chain peptides of 3 to 5 aa totaled 140, accounting for 87.0%, and sequences within 10 aa accounted for 90.1%. This indicates that the candidate umami peptides formed after doubanjiang fermentation were mainly low-molecular-weight oligopeptides. Residue composition was not randomly distributed. S, E, T, F, M, D, G, L, N, and V were high-frequency amino acids. Among the sequences, 88 contained D or E, 82 contained F, Y, or W, and 41 simultaneously contained acidic and aromatic residues. At the sequence termini, E, M, and Y were more frequently found at the N-terminus, whereas E, F, H, S, and Y were more frequently found at the C-terminus. This indicates that these peptides showed strong retention preferences during fermentative protein hydrolysis. Previous studies have shown that umami peptides usually occur in short-chain forms and that polar or acidic residues such as D, E, N, and Q play important roles in umami expression, whereas aromatic interactions, hydrogen bonding, and hydrophilicity are closely associated with receptor recognition.

From this perspective, short-chain sequences such as EESP, DYDS, ELDE, EVDQ, NYEE, QDEL, PSEE, and GVAEF, which contain both acidic sites and hydrophobic or aromatic termini, are structurally representative. A comparison with reported fermented-food-derived umami peptides showed that these exact sequences have not been clearly validated as umami peptides in previous studies. They were therefore regarded as newly screened doubanjiang-derived candidates in the present work, rather than as rediscovered known umami peptides. However, their structural features are consistent with many validated umami peptides from fermented foods. For example, soy sauce peptides LPEEV, AQALQAQA, and EQQQQ have reported umami thresholds of 0.43, 1.25, and 0.76 mmol/L, respectively [41]. Fermented broad bean paste-derived peptides such as DGF and HHYE have also been confirmed as low-threshold umami or umami-enhancing peptides, with thresholds of 0.37 and 0.21 mmol/L under different sensory conditions [62]. Dajiang-derived peptides have even lower reported thresholds, ranging from 0.02 to 0.14 mmol/L [63]. Compared with these known peptides, the 6 representative peptides confirmed in this study showed thresholds of 0.14 to 1.09 mmol/L, placing them within the common potency range of food-derived umami peptides. Their thresholds were also comparable to, or lower than, the reported recognition threshold range of MSG, which is often around 1 to 2 mmol/L in aqueous sensory systems [64]. This comparison indicates that the screened doubanjiang peptides are not only structurally plausible but also sensorially relevant. At the same time, nested sequences such as LDTSNIANQL, NQLDSTPRVF, LDTSNIANQLDSTPRVF, LDTSNTLNQLDSTPRLF, YLGGNPEVE, YLGGNPEVEFPE, YLGGNPEVEFPET, and SEEQNEGKSVLSGFSAE were simultaneously observed. This indicates that protein degradation in doubanjiang did not occur through one-step cleavage, but rather through continuous truncation and stagewise accumulation, which is also an important feature of the gradual enrichment of functional peptides in fermented systems.

In terms of flavor contribution, the potential roles of these candidate peptides in the umami of doubanjiang may be reflected at two levels. First, they may directly constitute the umami framework. Recent studies on fermented broad bean paste and Pixian doubanjiang have confirmed that multiple peptides derived from doubanjiang exhibit umami or umami-enhancing effects, and that some sequences have low thresholds. This indicates that peptides are not marginal components, but important constituents of the umami of doubanjiang. Second, they may provide taste compensation under salt-reduction conditions. Relevant studies have shown that umami and saltiness are the most prominent tastes of doubanjiang. Studies on saltiness-enhancing peptides have further shown that short-chain peptides, especially those carrying negatively charged side chains or terminal groups, can enhance saltiness perception or maintain salt intensity without introducing additional sodium ions. On this basis, the large number of D and E residues found in the candidate sequences in this study, together with acidic short peptides such as EESP, DYDS, NYEE, PSEE, QDEL, DEEH, and DSAGD, are more likely to exert umami-supporting and saltiness-synergistic effects in the high-salt doubanjiang matrix. In contrast, longer peptides such as LDTSNIANQLDSTPRVF, YLGGNPEVEFPET, and FYIGGNPEAEFPETQE are more likely to improve overall taste integrity by prolonging aftertaste, increasing taste body, and enhancing oral persistence. It can therefore be inferred that these candidate peptides not only participated in the formation of the umami of doubanjiang but also provided a molecular basis for maintaining taste fullness and reducing dependence on salt alone under salt-reduction conditions.

In addition, the contribution of these peptides should not be considered independently from other umami compounds in doubanjiang. During fermentation, glutamate, aspartate, IMP, GMP, organic acids, and peptides may coexist in the same taste matrix. Glutamate is the core ligand responsible for umami perception, while 5′-ribonucleotides such as IMP and GMP can strongly enhance glutamate-induced umami through the T1R1/T1R3 receptor. Umami peptides may further interact with MSG or nucleotide-related umami systems by providing additional hydrogen-bonding, electrostatic, and hydrophobic contacts in the receptor cavity, thereby enhancing receptor stabilization or modifying taste persistence. Therefore, the umami contribution of doubanjiang peptides is more likely to arise from a matrix-dependent interaction network rather than from a single peptide effect. This also explains why some peptides may support umami fullness, aftertaste continuity, or saltiness compensation even when their direct umami intensity is not dominant.

### 3.2. Preliminary Screening of Umami Peptides and Molecular Docking Analysis

After candidate umami peptides with UMPred-FRL probability values > 0.9 had been screened, a total of 161 small peptides were obtained for subsequent receptor docking analysis. To further evaluate their interaction characteristics with the umami recognition system, homology modeling and molecular docking were carried out using the T1R1/T1R3 heterodimer as the molecular recognition target. T1R1 and T1R3 together form the classical umami receptor, and their extracellular Venus flytrap domain is the core region for ligand capture, binding, and conformational response. It is therefore the receptor interface most commonly used in current molecular simulation studies of umami peptides. Previous studies have shown that peptides, amino acids, and other umami-related molecules can complete initial recognition within this domain, and that ligand binding is jointly stabilized by hydrogen bonding, electrostatic interactions, salt bridges, and hydrophobic contacts [12,55].

**Figure 1:**
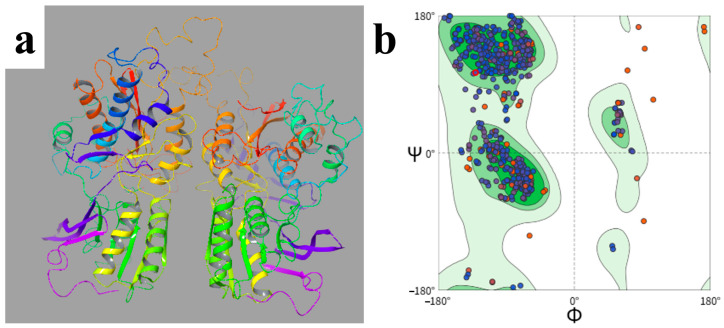
Homology modeling results of the umami receptor. (**a**) Homology model structure of the umami receptor T1R1/T1R3; (**b**) Ramachandran plot.

Given that the T1R1/T1R3 receptor consists of two subunits and that the two subunits differ to some extent in ligand recognition, the dimer was first separated into the T1R1 and T1R3 chains, and three-dimensional homology models were then established separately to reduce the risk of local mismatch and pocket distortion that might arise from direct overall modeling. After model construction, the distribution of backbone dihedral angles was stereochemically evaluated using the Ramachandran plot to determine whether the models had the geometric reasonableness required for docking. As shown in Figure 1a, the T1R1 model exhibited an overall closed conformation, whereas T1R3 remained relatively open. Together, they formed a recognition system that could accommodate umami ligands of different sizes, and the more open cavity of T1R3 was more favorable for the entry and extension of longer peptides. Recent receptor recognition studies have further indicated that T1R3 shows a greater tendency to accommodate longer or more complex peptides, whereas T1R1 makes a more prominent contribution to the recognition of small ligands such as dipeptides, tripeptides, and amino acids. Thus, the two subunits do not substitute for each other during umami ligand recognition, but instead participate jointly and complement each other.

The reliability of homology modeling is usually closely related to the similarity between the template and target sequences. Comparative modeling studies generally consider that when the sequence identity between the target protein and the template reaches about 30% or higher, a usable structural model can usually be obtained for binding-site analysis and molecular docking. Within the range of 25% to 50%, the model still retains good conformational reference value. On this basis, the sequence identities of T1R1 and T1R3 to the selected template were determined to be 34.34% and 33.55%, respectively, both within the acceptable range. Further Ramachandran results show that 97.7% of residues were distributed in allowed regions, with 87.7% in the favored region, 10.0% in additionally allowed regions, only 1.8% in generously allowed regions, and fewer than 0.5% in disallowed regions (Figure 1b). Because more than 90% of the residues fell within reasonable conformational space, the constructed models were considered to have high overall stereochemical quality and could provide a reliable structural basis for subsequent molecular docking screening and mechanistic analysis of the 161 candidate umami peptides.

Semi-flexible docking in the Schrödinger suite was used for molecular docking. All other parameters were kept at their default settings, and only the conformation with the lowest docking energy was retained. Statistical analysis of the 141 successfully docked candidate umami peptides showed that the sequences entering the receptor binding site were overwhelmingly dominated by short-chain oligopeptides (Table 3). Among them, 40 were tripeptides, accounting for 28.4%. A total of 84 were tetrapeptides, accounting for 59.6%. A total of 16 were pentapeptides, accounting for 11.3%. Only 1 hexapeptide was identified, accounting for 0.7%. This distribution indicates that receptor recognition did not favor long peptides, but instead preferred short-chain sequences with compact conformations and small steric occupancy. The docking scores ranged from −7.56 to −2.86, and the energy units given below are all in kcal/mol. The average score was −5.33, and the median was −5.30. Most scores were concentrated in the range from −6.0 to −4.0, with 106 peptides in total, accounting for 75.2%.

Another 30 peptides scored below −6.0, indicating that this group had better predict binding ability. The value of −6.0 was used as an operational screening cutoff rather than a statistical significance boundary. Among the 141 successfully docked peptides, 31 peptides, accounting for 22.0%, had docking scores lower than or equal to −6.0. This subset was therefore considered the high-score region of the present docking dataset. Because only the lowest-energy conformation was retained for each peptide, the docking scores were not generated from biological or technical replicates. Therefore, no statistical comparison such as ANOVA or multiple-comparison testing was performed among docking scores. The term significant difference was avoided for docking results, and the scores were interpreted only as relative ranking indicators. This treatment is consistent with the use of GlideScore as an empirical and semiquantitative scoring function for ligand ranking rather than as a direct experimental binding free energy [65,66]. Ranked by score, the top six peptides were EESP, SCPH, SSSGF, PDTE, SYH, and DYDS, with docking scores of −7.56, −7.30, −7.03, −6.85, −6.81, and −6.74, respectively. These six peptides were selected because they formed the highest-ranked continuous group and showed docking scores 1.41 to 2.22 kcal/mol lower than the overall mean score of all successfully docked peptides, which was −5.33 kcal/mol. The selection of six peptides was also a practical balance between computational ranking, sequence-type coverage, synthesis feasibility, and sensory workload. A larger number would have increased the cost and complexity of peptide synthesis and threshold validation, whereas a smaller number would have reduced structural representativeness. The selected peptides covered different sequence features, including acidic residue-rich peptides, Ser-rich peptides, His-containing peptides, and aromatic-terminal peptides. Therefore, they were used as representative high-affinity candidates for subsequent MM-GBSA analysis and sensory validation, rather than as peptides that were statistically different from all other docked sequences.

It should be noted that these six peptides were selected according to receptor binding priority rather than chromatographic abundance. In the present untargeted peptidomics workflow, the LC-MS peak area was used as a semi-quantitative signal to reflect relative detection intensity, but it was not equivalent to absolute peptide content because no isotope-labeled standards were used for each endogenous peptide. Therefore, the top six peptides according to docking score should not be interpreted as the most predominant peptides in doubanjiang. Instead, they represent high-affinity candidates with stronger predicted interaction with T1R1/T1R3. This selection strategy was consistent with the purpose of this study, which was to screen peptides with potential taste activity rather than to rank all peptides by abundance alone. To avoid overstatement, peptide abundance information was considered only as supporting evidence for detectability, whereas the final functional evaluation relied on docking, MM-GBSA analysis, threshold determination, and sensory addition validation [66,67].

In the Glide system, more negative scores generally indicate more favorable ligand binding. Therefore, these six peptides can be regarded as high-affinity representatives among the candidate sequences. Combined with recent receptor recognition studies, it can be seen that both T1R1 and T1R3 are capable of recognizing short-chain peptides. Among them, acidic and hydrophilic residues are more likely to participate in receptor recognition, whereas hydrogen bonding, electrostatic interactions, and aromatic interactions are important driving forces for stabilizing the complex.

At the sequence level, the top-ranked peptides were not simple repetitions of a single type, but showed strong structural complementarity. EESP, PDTE, and DYDS contained obvious acidic sites, which are favorable for polar anchoring and charge pairing. SCPH and SYH contained His, which may play a more active role in the hydrogen-bond network and local aromatic recognition. SSSGF combined Ser enrichment with a terminal Phe, thus retaining good flexibility while also possessing the potential for hydrophobic terminal insertion. Further comparison of the energy terms showed that SCPH ranked first and second in the whole set for Glide Gscore and Glide Hbond, respectively, indicating that its hydrogen-bond contribution was particularly prominent. PDTE ranked sixth in Glide Ecoul, suggesting a clear advantage in electrostatic interactions. SYH ranked second in Glide Emodel, indicating good conformational matching and overall complex stability. EESP ranked first in the overall docking score, indicating that it achieved a favorable balance among multiple interaction forces. On the basis of these results, the selection of EESP, SCPH, SSSGF, PDTE, SYH, and DYDS for subsequent mechanistic analysis was well supported. On the one hand, these six peptides formed the highest-scoring continuous tier in the current docking results and could represent the strongest predicted binding level in this study. On the other hand, they covered three peptide lengths, namely tripeptides, tetrapeptides, and pentapeptides, and also represented multiple sequence features, including acidic residue-enriched, sulfur- and heterocycle-containing, Ser-enriched, and aromatic terminal types. This was more favorable for comparing the interaction differences between different structural units and the umami receptor. In similar studies, a few top-scoring peptides with representative structural types are often retained from a larger candidate set and then subjected to further analysis in combination with molecular dynamics, binding free energy, and sensory validation. Therefore, these six peptides not only had strong docking advantages, but also showed good representativeness, and could be used as the core targets for subsequent investigation of the recognition mechanism of the umami receptor.

To place the screened peptides in the context of reported fermented-food-derived umami peptides, the present candidates were compared with known peptides from soy sauce, fermented broad bean paste, and other fermented matrices. The exact sequences EESP, SCPH, SSSGF, PDTE, SYH, and DYDS have not been clearly reported as validated umami peptides in previous fermented food studies. They were therefore considered newly screened doubanjiang-derived candidates. Even so, their structural and sensory features were comparable to those of known umami peptides. For example, LPEEV, AQALQAQA, and EQQQQ from soy sauce have been reported to show umami taste with thresholds of 0.43, 1.25, and 0.76 mmol/L, respectively [41]. Fermented broad bean paste-derived peptides, including PKALSAFK, NKHGSGK, SADETPR, EIKKAALDANEK, DALAHK, LDDGR, and GHENQR, have also been confirmed as umami-related peptides through separation, mass spectrometry, sensory evaluation, and receptor interaction analysis [62]. Compared with these reported peptides, the six representative peptides in this study showed thresholds of 0.14 to 1.09 mmol/L, indicating that their taste potency was within the common range of validated food-derived umami peptides [62,68]. More importantly, the linkage from identification to actual taste contribution was built through a stepwise evidence chain rather than by prediction alone. Peptidomics first confirmed that these sequences were present in doubanjiang. UMPred-FRL then prioritized candidates with high umami potential. Molecular docking and MM-GBSA further suggested possible interaction with T1R1/T1R3. Threshold determination and peptide addition experiments finally verified their sensory effects in the real doubanjiang matrix. This integrated evidence supports their potential contribution to taste, while also showing that their main role in doubanjiang may include saltiness synergy and taste-structure remodeling, rather than direct umami enhancement alone.

### 3.3. Molecular Mechanism Analysis of Six Umami Peptides and Their Receptor Proteins

**Figure 2:**
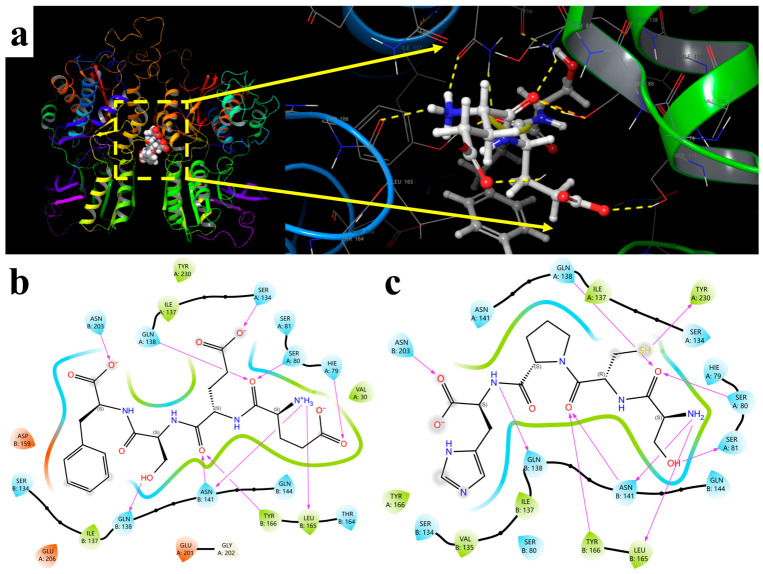

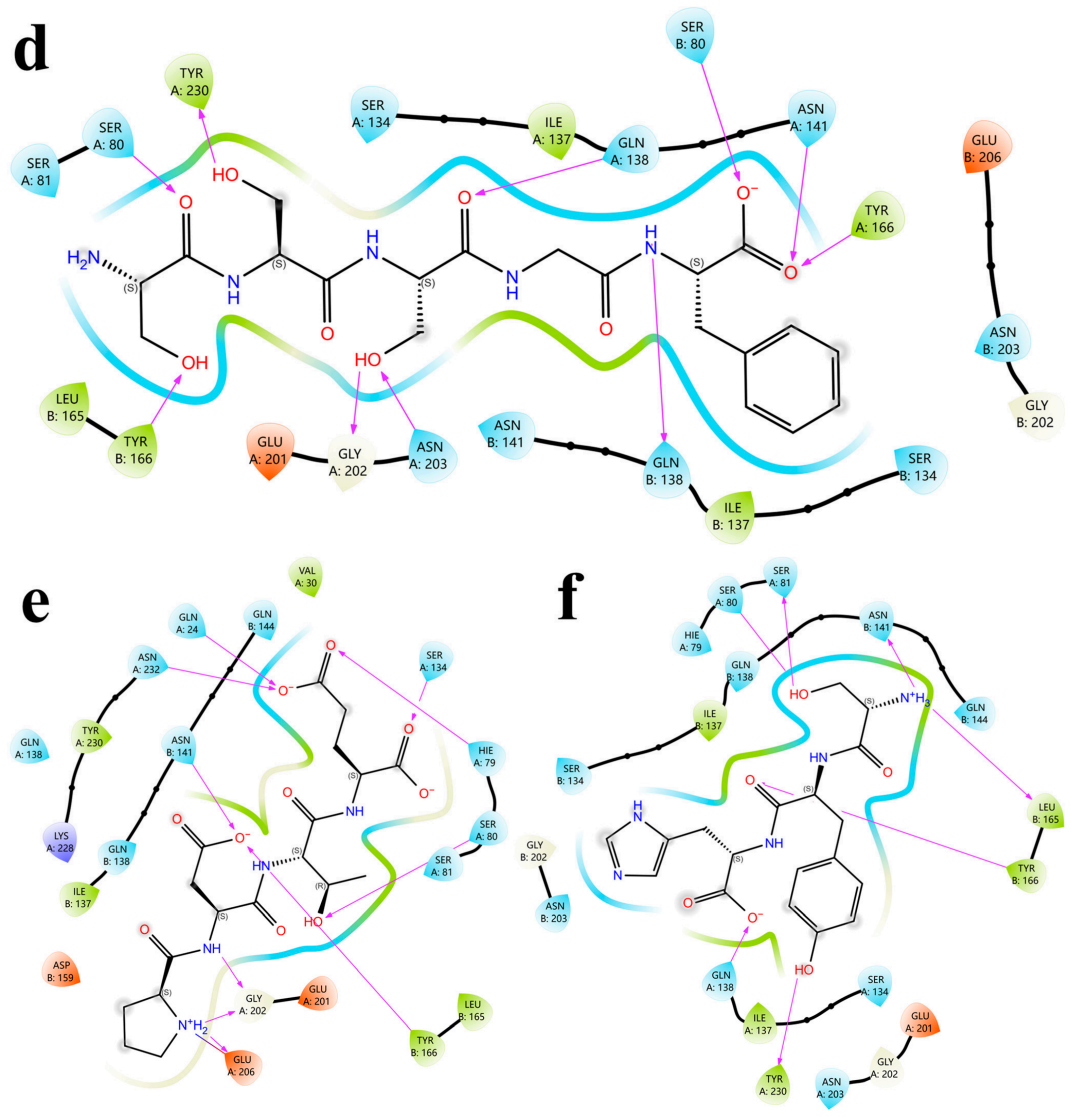

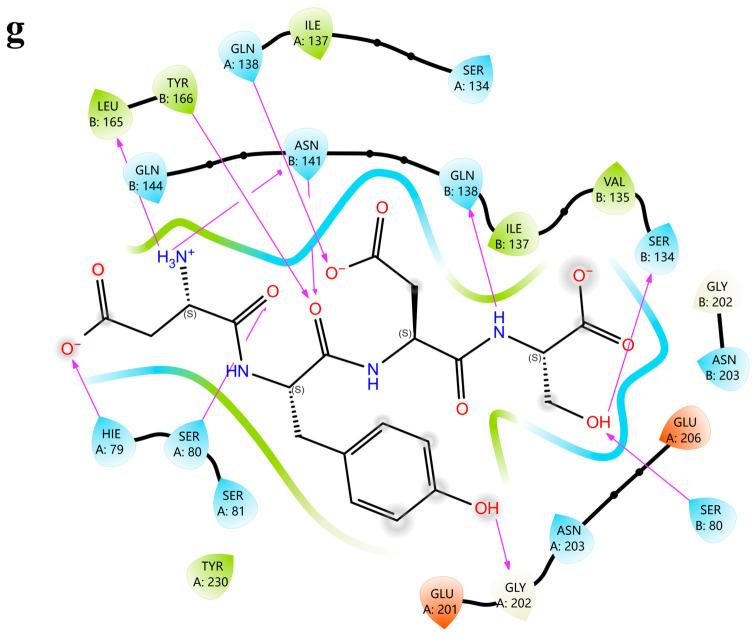
Schematic representation of molecular docking. The left panel shows the overall view, and the right panel shows the close-up intermolecular interaction pattern (**a**). Hydrogen bonds are shown in blue, halogen bonds in purple, salt bridges in red, aromatic hydrogen bonds in cyan, π–π stacking interactions in blue, and π–cation interactions in green. The 2D binding mode diagrams, based on the docking-derived binding modes of T1R1/T1R3 with EESP, SCPH, SSSGF, PDTE, SYH, and DYDS, are labeled (**b**–**g**).

As shown by the 2D interaction diagram, this umami peptide mainly bound within the VFT recognition cavity of the T1R1/T1R3 umami receptor heterodimer (Figure 2a). The stable binding of EESP did not depend on a single interaction force but was jointly maintained by multipoint hydrogen-bond anchoring and local hydrophobic burial. Polar recognition was central to the formation of this complex. Ser A134, Gln A138, Ser A80, and Hie A79 on the T1R1 chain, together with Asn B203, Asn B141, and Gln B138 on the T1R3 chain, jointly participated in the directional recognition of carboxyl or amide oxygens, thereby effectively fixing the carboxyl groups at both ends of the ligand. At the same time, Ile A137, Tyr A230, and Val A30 on the T1R1 chain, as well as Tyr B166 and Leu B165 on the T1R3 chain, formed a hydrophobic confinement environment around the ligand. This not only restricted excessive swinging of the peptide chain but also facilitated the insertion of aromatic side chains and cyclic structures into the pocket (Figure 2b). These results indicate that the molecular recognition mechanism between this umami peptide and the receptor was mainly characterized by a hydrogen-bond network providing binding specificity, while hydrophobic interactions maintained conformational stability. Their synergy determined the residence and signal-triggering ability of the peptide in the receptor pocket.

As shown in Figure 2c, the terminal carboxyl group of SCPH was first directionally fixed by Asn B203. The backbone amide and carbonyl groups then formed a continuous hydrogen-bond network with Gln B138, Hie A79, Ser A80, Asn B141, and Ser A81, allowing the peptide chain to maintain a relatively stable extended conformation in the binding cavity. The sulfhydryl-containing side chain was located in the middle of the pocket and was adjacent to Gln A138, Ile A137, and Tyr A230, indicating that this region not only provided polar recognition but also played a role in local spatial confinement. Tyr A166, Val B135, Ile B137, Tyr B166, and Leu B165 formed a hydrophobic environment around the peptide chain, which helped restrict conformational fluctuation and improve complex stability. Overall, the recognition mechanism of this peptide was not dominated by a single residue. Instead, binding specificity was established by polar residues such as Asn, Gln, Ser, and Hie, whereas hydrophobic residues such as Tyr, Ile, Val, and Leu provided conformational support, together driving the stable residence of the ligand in the receptor pocket.

The left terminal carboxyl group of SSSGF was first directionally recognized by Asn B203. The left-side carbonyl group and adjacent polar groups then formed stable coordination with Ser A80 and Tyr A230 (Figure 2d). After entering the middle of the pocket, the backbone carbonyl and amide groups were constrained by Gln A138 and Gln B138, respectively, allowing the peptide chain to maintain a relatively extended conformation. The right terminal carboxyl group was another key anchoring site and mainly formed polar interactions with Ser B80, Asn A141, and Tyr A166, thereby enhancing terminal positioning. At the same time, Ile A137, Ile B137, Leu B165, and Tyr A166 formed a hydrophobic burial environment around the peptide chain, which not only restricted ligand fluctuation but also helped improve the overall stability of the complex. The recognition of this peptide by the umami receptor was therefore not dominated by a single residue, but by polar residues such as Asn, Gln, Ser, and Tyr that provided binding specificity, together with hydrophobic residues such as Ile and Leu that maintained spatial support, jointly promoting effective receptor recognition of the peptide ligand.

The umami peptide PDTE mainly bound within the VFT recognition cavity of the T1R1/T1R3 umami receptor, and its stability arose from the synergy between polar anchoring and hydrophobic confinement. The left terminal carboxyl group of the peptide was preferentially recognized by Gln A24 and Asn A232. The central carbonyl and amide groups continued to form continuous hydrogen bonds with Asn B141 and Gln B138, thereby fixing the backbone conformation. The right terminal carboxyl group interacted with Ser A134, Hie A79, and Ser A80, indicating that this region was another key anchoring site. The hydroxyl group on the peptide chain also participated in polar recognition near Ser A81 and Tyr B166, which helped strengthen local coordination. The protonated amino group at the N-terminus was located near Gly A202, Glu A201, and Glu A206, suggesting that the terminal positive charge might contribute to electrostatic stabilization (Figure 2e). Tyr A230, Val A30, Ile B137, and Leu B165 around the pocket formed a hydrophobic boundary that restricted peptide chain fluctuation and increased the compactness of the complex.

The hydroxyl and carbonyl groups at the upper end of SYH were first directionally recognized by Ser A80, Ser A81, and Gln B138, indicating that this polar cluster was the primary fixation region after ligand entry into the pocket (Figure 2f). The positively charged terminal amino group further interacted with Asn B141, providing additional charge stabilization at the right end of the peptide. The central carbonyl and carboxyl groups of the molecule formed coordination with Gln A138 and adjacent polar residues, respectively, which helped maintain the extended backbone conformation. Although no strong ionic interaction was shown in the region containing the aromatic side chain, it was surrounded by Tyr B166, Leu B165, and Ile B137, forming a hydrophobic boundary that restricted conformational fluctuation and increased complex compactness. Another noteworthy site was Tyr A230, which was adjacent to the phenolic hydroxyl group, suggesting that this residue might participate in local polar recognition and spatial correction. Overall, the recognition mechanism of this peptide was characterized by polar residues such as Ser, Asn, and Gln providing binding specificity, while hydrophobic residues such as Tyr, Leu, and Ile maintained spatial support. Together, these interactions determined the stable residence of the complex.

The left terminal carboxyl group of DYDS formed directional recognition with Hie A79, and the adjacent amide carbonyl group was further constrained by Ser A80. Two central carbonyl groups and carboxyl oxygens formed continuous interactions with Gln A138, Tyr B166, and Asn B141, allowing the peptide chain to maintain a relatively stable extended conformation. The right terminal amide NH was recognized by Gln B138, whereas the terminal carboxyl group formed a key coordination interaction with Ser B134. At the same time, the positively charged amino group at the N-terminus was located near Gln B144 and Leu B165, and the hydroxyl group on the right side was close to Ser B80, indicating that this peptide was jointly regulated in the pocket by charge interactions, a hydrogen-bond network, and the local hydrophobic environment. Overall, the key interacting residues included Hie A79, Ser A80, Gln A138, Asn B141, Gln B138, Gln B144, Ser B134, Ser B80, Tyr B166, and Leu B165, among which hydrogen bonding and electrostatic interactions determined binding specificity, whereas hydrophobic residues were responsible for conformational support (Figure 2g).

In summary, the docking results of umami peptides with the T1R1/T1R3 receptor showed that recognition mainly occurred in the extracellular VFT domain of the receptor protein. Its molecular basis was not driven by a single interaction force, but by the combined effects of hydrogen-bond networks, electrostatic attraction, and hydrophobic confinement. Polar sites containing carboxyl, carbonyl, amide, and hydroxyl groups usually formed directional coordination first with residues such as Ser, Asn, Gln, and His, thereby effectively anchoring both peptide termini and key backbone sites. This was the core source of specificity in complex formation. At the same time, hydrophobic residues such as Tyr, Ile, Leu, and Val formed a local burial environment around the peptide chain, which restricted ligand fluctuation, maintained extended or semi-extended conformations, and improved residence stability within the binding cavity. For peptides containing positively charged amino groups or ionizable side chains, nearby sites such as Glu and Gly could further provide charge stabilization or auxiliary spatial correction, thereby enhancing overall binding compactness. These interaction features were broadly consistent with experimentally supported binding information for the umami receptor. Site-directed mutagenesis and chimeric receptor studies have shown that the human T1R1 VFT domain is central to L-Glu and nucleotide-dependent umami recognition. Residues reported to be critical for L-Glu binding include Thr149, Ser172, Asp192, Tyr220, and Glu301, while His71, Arg277, Ser306, and His308 have been linked to IMP binding and umami synergism. Further mutagenesis work identified additional T1R1 VFT residues that modulate acidic amino acid recognition and species-dependent ligand specificity, including Ser148, Arg151, Glu174, Ala170, Ala302, and Asp435. More recent peptide-oriented receptor studies also reported conserved T1R1 VFT residues such as Asn150, Arg151, Thr154, Ser217, Gln222, and Ser248 as important sites for peptide recognition [69,70]. In comparison, the residues identified in the present docking analysis, including Ser, Asn, Gln, His, Tyr, Ile, Leu, Val, and Glu residues, were located in the same extracellular recognition region and belonged to similar functional residue classes. They provided hydrogen bonding, charge pairing, aromatic or hydrophobic packing, and spatial restriction. Because the present receptor was a homology model, residue numbering could not be directly equated with all experimentally validated human T1R1 sites. However, the overlap in domain location and interaction chemistry supports the reliability of the predicted binding mode. It also suggests that the doubanjiang peptides may occupy or approach the canonical umami-recognition region rather than binding randomly on the receptor surface. Accordingly, the interaction mechanism between umami peptides and the umami receptor protein can be summarized as follows: polar residues dominate recognition initiation and site locking, whereas hydrophobic residues are responsible for conformational support and spatial adaptation. Their synergy determines the stable residence of the ligand in the T1R1/T1R3 pocket and its subsequent receptor activation ability. This pattern indicates that the activity expression of umami peptides essentially depends on the balance between polar anchoring capacity and hydrophobic matching.

### 3.4. Analysis of MM-GBSA Binding Energies of Six Umami Peptides

As shown in Table 4, the six peptides showed different predicted binding stability with the receptor according to MM-GBSA calculation. Because MM-GBSA values were obtained from endpoint energy estimation rather than replicated experimental measurements, the term significant difference was avoided here unless independent computational or experimental replicates were used for statistical testing. The total binding free energy ranked from low to high as SCPH, SYH, SSSGF, DYDS, EESP, and PDTE, with values of −81.21, −79.99, −73.34, −49.39, −46.12, and −37.30, respectively. All subsequent energy values are expressed in kcal/mol. These results indicate that the complexes formed by SCPH and SYH were the most stable, whereas PDTE showed the weakest overall binding advantage. This ranking was not fully consistent with the Glide docking score ranking, in which EESP showed the most favorable docking score. Such inconsistency is reasonable because the two metrics describe different levels of molecular recognition. Docking score is an empirical and rapid scoring function. It is mainly used to predict binding pose and prioritize candidates during virtual screening. It does not fully account for receptor relaxation, desolvation penalty, or the energetic cost of polar group burial. In contrast, MM-GBSA is a force-field-based endpoint method that re-evaluates the docked complex by combining molecular mechanics energy with polar and nonpolar solvation terms. Therefore, acidic peptides such as EESP and PDTE may obtain favorable docking scores through strong hydrogen bonding or electrostatic anchoring, but their final MM-GBSA energies may be partly weakened by larger polar solvation penalties. SCPH and SYH showed more favorable total binding free energies, probably because their moderate polarity, aromatic or heterocyclic residues, and better hydrophobic fitting produced a more balanced energetic profile. For this reason, docking scores were used in this study for preliminary candidate prioritization and pose selection, whereas MM-GBSA was considered more informative for comparing relative binding stability among the six selected peptides within the same receptor system. However, MM-GBSA was still interpreted as computational supporting evidence rather than definitive proof of taste activity. The final functional judgment was therefore based on the combined evidence from docking, MM-GBSA, threshold determination, and sensory addition validation [71,72].

Further analysis of the individual energy terms showed that the vdW term of each peptide was significantly negative, ranging from −43.19 to −59.92, and represented the major favorable contribution shared by all systems. The Lipo term was stably distributed between −8.47 and −12.29, suggesting that hydrophobic burial also made a sustained contribution to complex stability. The Hbond term was concentrated between −5.16 and −7.15, indicating that hydrogen-bond networks were generally present among different sequences, although their individual variation was limited and was more closely related to fine regulation of binding conformation. In contrast, Coulomb and Solv GB showed a marked coupled compensation pattern. EESP, PDTE, and DYDS each contained two acidic residues, and the absolute values of both Coulomb and Solv GB increased significantly, indicating that these sequences had a stronger tendency for polar recognition, but also incurred a higher solvation compensation cost, which ultimately weakened the gain in total free energy. By contrast, SCPH, SYH, and SSSGF had a lower polar burden, but achieved more favorable vdW, Lipo, and Packing synergy, and therefore showed better total binding free energies. These results suggest that the high-affinity binding of umami peptides to the receptor does not depend solely on electrostatic attraction, but rather on the overall balance among polar recognition, hydrophobic fitting, and conformational matching.

The energy differences can be further explained by the sequence characteristics of the six peptides. Although SCPH and SYH do not contain Asp or Glu, both are short, compact peptides and contain aromatic or heterocyclic residues. In SCPH, Pro can restrict backbone freedom and reduce the conformational rearrangement cost during binding, whereas His possesses weak basicity, aromaticity, and hydrogen-bond donor and acceptor properties, allowing relatively flexible local recognition within the pocket. In SYH, Tyr and His are favorable for providing π-related interactions and hydrophobic fitting, whereas Ser contributes hydroxyl-based hydrogen-bonding sites. These two peptides therefore appear to achieve high affinity through moderate polar anchoring together with hydrophobic registration, rather than through strong charge-driven interactions. The result for SSSGF was also representative. This sequence contains three consecutive Ser residues, which can improve hydrogen-bond accessibility and interfacial wetting adaptability. Gly provides the necessary flexibility, whereas the terminal Phe markedly enhances vdW interactions and hydrophobic burial. Therefore, it maintained a relatively low total binding free energy even in the absence of acidic residues. In comparison, EESP, PDTE, and DYDS are more consistent with the general view in the literature that Asp and Glu are key active sites in umami peptides. However, the present data show that an increase in the number of acidic sites does not automatically translate into better binding. The reason is that continuous or high-density acidic side chains in short peptides can markedly enhance peptide affinity for the aqueous phase, thereby amplifying the polar compensation term during binding and partially offsetting the local advantage gained from receptor recognition. PDTE is a typical example. Among the six peptides, it showed the strongest Hbond contribution, but its Lipo and Packing terms were weaker, and the polar compensation effect was the most pronounced. As a result, its total free energy was the highest. Because DYDS contains Tyr, it obtained stronger vdW support than EESP and PDTE, and its overall stability was therefore slightly improved.

Overall, the present results support a balanced recognition mechanism. Efficient binding of umami peptides does not require simply increasing the number of acidic residues. Instead, synergy is needed among acidic anchoring sites, hydroxyl-type hydrogen-bonding sites, aromatic or hydrophobic side chains, and backbone conformational preorganization. Some studies have likewise shown that Asp and Glu are the most common umami-active sites in peptides with lengths of 4 to 7 residues, but that hydrogen-bond networks, hydrophobic contacts, and local spatial adaptation are also indispensable. On the other hand, increased exposure of hydrophobic residues is often associated with enhanced bitterness. Therefore, sequences that truly favor umami expression are usually not purely highly polar or highly hydrophobic types, but short peptide structures that achieve a balance between receptor affinity and sensory purity.

### 3.5. Verification Analysis of the Effects of Representative Umami Peptides on the Umami of Doubanjiang

As shown in Table 5, all six umami peptides were short-chain oligopeptides with lengths of 3 to 5 amino acid residues and relative molecular masses ranging from 405.1648 to 510.1962 Da, indicating that they fell within the typical range of small bioactive umami peptides. In terms of taste characterization, umami was dominant in all peptides, but it was not the only taste attribute. EESP, PDTE, and DYDS showed certain sour-umami characteristics, whereas SSSGF, SYH, and SCPH exhibited milder or rounder umami profiles, accompanied by slight bitterness, bitter–astringency, or drying sensations. These results indicate that the sensory expression of umami peptides was clearly complex. Threshold results further showed that the umami thresholds of the six peptides ranged from 0.14 to 1.09 mmol/L and were ranked as SCPH < SYH < SSSGF < EESP < DYDS < PDTE. In general, a lower threshold indicates that a peptide can be perceived at a lower concentration and therefore has stronger umami activity. Accordingly, SCPH and SYH showed relatively better umami recognition capacity, whereas PDTE had the highest threshold, indicating a relatively weaker contribution to umami. Combined with the sequence characteristics, the intensity of umami did not appear to depend solely on the number of acidic residues. Instead, it was more likely related to molecular mass, sequence length, and the synergistic combination of acidic, polar, and hydrophobic residues. This is consistent with the view that the taste characteristics of umami peptides are jointly regulated by sequence composition and structure.

**Figure 3:**
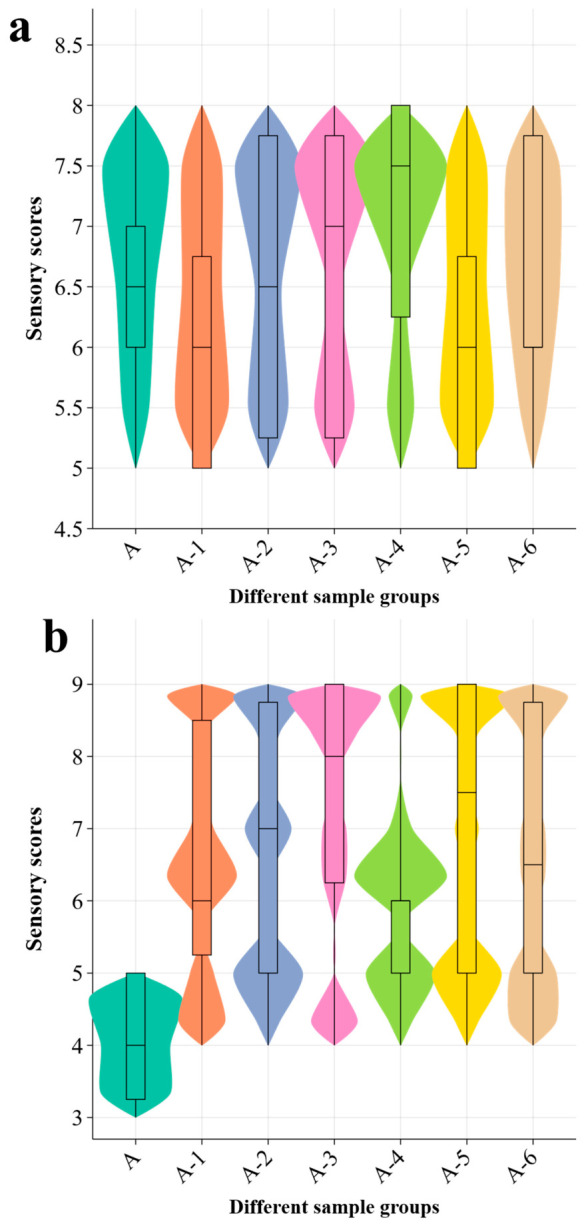
Comparison of the umami (**a**) and saltiness (**b**) of doubanjiang before and after the addition of representative umami peptides.

As shown in Figure 3, after addition of the respective umami thresholds of the six peptides, the saltiness response of the samples increased consistently, whereas the umami response showed clear divergence. All sensory data are expressed as mean ± SD (n = 10). Because the same trained panel evaluated all samples, differences among the control and peptide-added groups were analyzed by repeated-measures one-way ANOVA, followed by Holm–Bonferroni adjusted paired comparisons against the control. In terms of umami, the control sample scored 6.40 ± 0.97. PDTE increased the score to 7.00 ± 1.25, with an increase of 9.38%, and showed the strongest apparent umami-enhancing effect. SSSGF increased the score to 6.60 ± 1.26, with an increase of 3.13%. SCPH and DYDS both increased the score to 6.50 ± 1.27 and 6.50 ± 1.18, with increases of 1.56% for both. In contrast, EESP and SYH decreased the scores to 6.10 ± 1.20 and 6.00 ± 1.05, with decreases of 4.69% and 6.25%, respectively. However, the overall umami difference was not significant among groups (F (6,54) = 0.775, *p* = 0.593), and none of the peptide-added samples differed significantly from the control after post hoc adjustment (adjusted *p* > 0.05).

In terms of saltiness, the control sample scored 4.10 ± 0.88. All six peptide-added samples showed significant enhancement, with increases ranging from 43.90% to 78.05%. Among them, SSSGF showed the most pronounced increase in saltiness, with the score rising to 7.30 ± 2.00, corresponding to an increase of 78.05%. SYH and SCPH increased to 7.00 ± 2.05 and 6.80 ± 1.93, with increases of 70.73% and 65.85%, respectively. DYDS and EESP increased to 6.60 ± 2.07 and 6.50 ± 1.96, with increases of 60.98% and 58.54%, respectively. Although PDTE showed the smallest increase, its saltiness score still reached 5.90 ± 1.37, corresponding to an increase of 43.90%. Repeated-measures ANOVA confirmed a significant treatment effect for saltiness (F(6,54) = 3.260, *p* = 0.008). Post hoc comparison with the control further showed that all peptide-added samples significantly increased saltiness (EESP, adjusted *p* = 0.019; SCPH, adjusted *p* = 0.006; SSSGF, adjusted *p* = 0.006; PDTE, adjusted *p* = 0.019; SYH, adjusted *p* = 0.019; DYDS, adjusted *p* = 0.019). Overall, the average saltiness score of the treated groups reached 6.68, which was 63.01% higher than that of the control, whereas the average umami score increased by only 0.78%. These results indicate that, under the high-salt and strongly fermented background of doubanjiang, the main role of the six umami peptides was not reflected in the simultaneous amplification of umami but was more concentrated in saltiness enhancement and taste-structure remodeling.

Previous studies have shown that, in doubanjiang and other fermented soybean products, proteins are continuously degraded under microbial and enzymatic action to form multiple taste-active substances, including amino acids, nucleotides, organic acids, and umami peptides. Peptides with both umami-enhancing and saltiness-enhancing functions have also been reported in fermented doubanjiang. Therefore, sensory output in complex matrices is often governed by multicomponent synergy rather than by the linear contribution of a single peptide. It can thus be seen that the effects of the six representative umami peptides in doubanjiang were clearly sequence-dependent. PDTE was more suitable as an umami-enhancing peptide. SSSGF, SYH, and SCPH were more suitable as saltiness-synergistic peptides. The application value of EESP and DYDS was more likely to lie in complex taste regulation rather than in simple umami enhancement.

Taken together with the sensory validation results and the preceding mechanistic analysis, the designation of umami peptides appears to reflect their potential taste-active properties rather than an inevitable umami-enhancing effect in every food system. Previous studies have shown that umami peptides can bind to the T1R1/T1R3 receptor, but their actual sensory output is not determined by receptor affinity alone [73]. It is also jointly regulated by food matrix composition, the background of endogenous umami substances, and interactions among different taste pathways [74]. In complex foods, umami substances themselves may enhance saltiness, suppress bitterness, and produce different perceptual outcomes as the system composition changes [75].

In the present study, the addition of six representative umami peptides to doubanjiang did not generally lead to a simultaneous increase in umami. Instead, the effect was more prominently reflected in saltiness enhancement, and this phenomenon is not contradictory. Doubanjiang is a complex matrix characterized by high salt content, strong fermentation, and highly coupled components. In this system, amino acids, nucleotides, organic acids, and peptides jointly participate in taste construction. After exogenous peptides enter the matrix, they do not simply add their own umami to the system [12,76]. Rather, they reshape the overall taste profile by altering the existing taste balance and affecting salt release and salt perception intensity. Studies on doubanjiang and related fermented soybean products have likewise shown that umami peptides, amino acids, and organic acids formed during fermentation jointly determine the final umami expression, and that some peptides can simultaneously enhance umami and saltiness. This indicates that the functional role of peptides is clearly matrix-dependent. More specifically, short-chain peptides, especially those containing acidic residues, may not only participate in umami receptor recognition but may also be involved in salt perception through salt-related pathways or ionic environment regulation. Existing reviews have indicated that short-chain peptides carrying negatively charged side chains or terminal groups can exhibit saltiness-enhancing properties in high-sodium systems, and that their mechanisms are related to pathways such as CaSR, TRPV1, and TMC4. Therefore, the evaluation of umami peptides should not remain limited to sequence prediction, molecular docking, or threshold determination in aqueous solution [74,77,78]. Greater emphasis should be placed on real food systems, in combination with sensory results, effective concentrations, and overall taste-profile changes in specific matrices. In other words, the functional positioning of umami peptides may differ among foods. In some systems, they mainly act as umami enhancers. In others, they are more likely to contribute to saltiness synergy, bitterness masking, or flavor rounding. This is also the fundamental reason why their application and development must be based on actual sensory performance in foods.

## 4. Discussion

### 4.1. Structure–Docking Correlation of Successfully Docked Umami Peptides

To better explain the relationship between peptide sequence features and receptor recognition, the 141 successfully docked peptides were further analyzed using the docking data. These peptides were mainly short-chain sequences, with 40 tripeptides, 84 tetrapeptides, 16 pentapeptides, and only 1 hexapeptide. The docking scores ranged from −7.56 to −2.86 kcal/mol, with a mean value of −5.33 kcal/mol. A total of 31 peptides showed docking scores lower than or equal to −6.00 kcal/mol, indicating relatively stronger predicted binding. Among them, EESP, SCPH, and SSSGF were the only peptides with scores lower than −7.00 kcal/mol. EESP, SCPH, SSSGF, PDTE, SYH, and DYDS formed the highest-scoring continuous group, with docking scores from −7.56 to −6.74 kcal/mol. Their average docking score was much lower than that of the whole docked peptide set, −7.05 versus −5.33 kcal/mol, which supports their selection as representative peptides for further validation.

Correlation analysis further indicated that receptor binding was not determined by peptide length alone. The affinity index, calculated as the negative value of the docking score, showed a positive correlation with charged residue number (Spearman rho = 0.251, *p* = 0.003), but a negative correlation with hydrophobic residue number (rho = −0.234, *p* = 0.005). The absolute hydrogen-bond contribution was positively correlated with Ser number (rho = 0.355, *p* < 0.001) and polar residue number (rho = 0.347, *p* < 0.001), suggesting that polar residues helped stabilize peptide recognition through hydrogen bonding. The Coulomb contribution was more closely related to basic residue number (rho = 0.427, *p* < 0.001), while vdW contribution increased with peptide length (rho = 0.499, *p* < 0.001) and aromatic residue number (rho = 0.266, *p* = 0.001). These trends are consistent with previous reports that umami peptides are commonly stabilized in the T1R1/T1R3 binding cavity through hydrogen bonds, electrostatic interactions, hydrophobic contacts, and local spatial fitting.

The six selected peptides also reflected this balanced recognition pattern. EESP, PDTE, and DYDS contained acidic residues and showed strong electrostatic contribution. SCPH, SSSGF, and SYH contained more polar residues, with SCPH showing the strongest hydrogen-bond contribution among the six peptides. SYH had the most favorable Glide Emodel value, indicating better overall pose stability and conformational matching. SSSGF combined multiple Ser residues with terminal Phe, which may explain its simultaneous hydrogen-bonding capacity and vdW support. These results suggest that the predicted receptor recognition of doubanjiang umami peptides depends on a coordinated structural pattern rather than on a single residue type. Charged and polar residues favor initial anchoring and hydrogen-bond formation, while aromatic or hydrophobic residues provide local packing and spatial adaptation. This structure–docking relationship supports the later sensory finding that different peptides did not contribute to taste in the same way. Some peptides were more closely related to direct umami support, while others were more likely to participate in saltiness synergy or taste-structure remodeling in the doubanjiang matrix.

### 4.2. Subunit Specific Interaction Pattern of T1R1 and T1R3

Based on the residue annotations in the 2D interaction maps of the six representative peptide complexes, 72 distinct receptor residue contacts were recorded. Among them, 38 contacts were assigned to T1R1 and 34 contacts were assigned to T1R3, corresponding to 52.8% and 47.2% of the total interactions. This indicates that peptide recognition was not controlled by a single subunit. T1R1 showed a slightly higher overall contact contribution, especially for EESP, SSSGF, and PDTE. The T1R1 to T1R3 contact ratios of these complexes were 7:5, 6:5, and 11:5, respectively. PDTE showed the strongest T1R1 preference, which was mainly related to multiple contacts with Gln A24, Asn A232, Ser A134, Hie A79, Ser A80, Ser A81, Glu A201, Gly A202, and Glu A206. By contrast, DYDS showed a clear T1R3 preference, with a T1R1 to T1R3 contact ratio of 3:7. SYH also showed a weak T1R3 bias, with a ratio of 4:5. SCPH was nearly balanced, with 7 contacts on each subunit. These results suggest that T1R1 mainly provides polar anchoring and local charge stabilization for several acidic peptides, while T1R3 contributes important complementary recognition sites for terminal fixation, aromatic packing, and cavity adaptation. This balanced but peptide-dependent subunit contribution is consistent with the reported view that umami peptides are recognized by the T1R1/T1R3 heterodimer through hydrogen bonding, electrostatic interaction, hydrophobic contact, and spatial fitting within the VFT region [66,79]. Therefore, the receptor recognition of doubanjiang peptides should be regarded as a cooperative T1R1/T1R3 process, rather than a T1R1-only or T1R3-only event.

### 4.3. Salt Reduction, Preservation, and Health Relevance

Salt reduction in doubanjiang should be understood as a controlled reformulation strategy, not as a simple removal of NaCl. Salt still has to protect the product during fermentation and storage. It helps regulate osmotic pressure, lower water activity, suppress undesirable microorganisms, and shape enzyme activity and flavor formation. If the salt level is reduced too strongly, the fermentation ecology may shift. This may weaken flavor, increase microbial risk, or promote the accumulation of undesirable metabolites such as biogenic amines [8,80]. For this reason, the salt-reduced system used in this study was designed to keep a preservation function while reducing sodium pressure. Its feasibility should be judged together with microbial counts, pH, water activity, amino acid nitrogen, and biogenic amine levels in future process validation. From a health perspective, this direction is meaningful because excessive sodium intake is closely related to hypertension and cardiovascular risk. A moderate reduction in sodium in a high-salt condiment can help lower dietary sodium exposure, especially when the product is consumed frequently. The practical goal is therefore not salt elimination. It is a safer balance. Enough salt is retained to maintain the typical fermented character and preservation stability of doubanjiang, while peptide-based taste compensation helps reduce dependence on excessive NaCl.

### 4.4. Matrix Interactions Among Bioactive Compounds and Umami Perception

The sensory effect of umami peptides should be interpreted within the whole food matrix rather than from the peptide sequence alone. In doubanjiang, peptides coexist with amino acids, organic acids, sugars, nucleotides, phenolic compounds, salts, and volatile metabolites. These compounds may interact through addition, suppression, masking, or cross-modal balance. Therefore, the final umami response is not necessarily proportional to the amount or predicted activity of 1 peptide. This also explains why some peptides in this study enhanced saltiness more clearly than umami. Similar matrix-dependent behavior has been reported for umami substances, which can modulate sweetness, saltiness, sourness, and bitterness in complex foods. Such interactions may improve or weaken hedonic response depending on the surrounding matrix. This principle is also relevant to functional fruit and vegetable beverages. The recent study on Spirulina-enriched fruit and vegetable juices in *Nutrients* showed that consumer preference depended on the balance between bioactive enrichment and sensory acceptance, rather than nutritional value alone. In the present doubanjiang system, umami peptides may therefore act as taste modulators [81,82,83]. Their practical value lies not only in direct umami enhancement but also in rebuilding taste balance under salt-reduction conditions through interactions with other taste-active and bioactive compounds.

### 4.5. Practical Applications of the Findings

The present findings provide a practical route for salt reduction in traditional doubanjiang. The value of these peptides should not be understood only as new flavor molecules. Their larger value lies in how they can guide product design, process control, and quality evaluation. Recent studies on Pixian doubanjiang have also shown that peptide-based screening can identify umami active sequences and clarify their interaction with T1R1/T1R3. This supports the use of peptide markers for the rational improvement of fermented condiments.

A direct application is the development of salt-reduced doubanjiang with less sensory loss. Sodium reduction in bean-based fermented foods is difficult because salt is tied to microbial stability, enzyme activity, texture, and flavor formation. Excessive reduction may cause microbial risk, flavor weakening, and adverse metabolite accumulation. A recent review on bean-based fermented foods emphasized that sodium reduction must be supported by a clear mechanism, rather than by simple salt removal. In this context, SSSGF, SYH, and SCPH are useful candidate peptides because they showed stronger saltiness synergy in the doubanjiang matrix. They may help maintain perceived saltiness when NaCl is moderately reduced. PDTE showed stronger umami enhancement. It may be more suitable for rebuilding the savory backbone. EESP and DYDS may be used as taste-balancing candidates, rather than single-purpose umami enhancers.

These results also offer a process-oriented application. The six peptides can be used as endogenous markers to evaluate whether fermentation has generated a favorable taste-active peptide profile. Instead of adding large amounts of external flavor enhancers after fermentation, manufacturers may adjust raw material ratios, koji preparation, starter activity, salt level, and aging time to promote the formation of desirable peptides. This is important for clean label development. It also fits the logic of traditional fermentation. The product keeps its own flavor identity, while the fermentation process is guided by molecular evidence.

Another practical application is quality control. Doubanjiang quality is often judged by color, aroma, saltiness, umami, and overall taste body. These indicators are useful but can be subjective. The peptide evidence obtained here can be converted into measurable targets. For example, PDTE may be monitored as a potential umami support marker. SSSGF, SYH, and SCPH may be monitored as saltiness synergy markers. Such markers could be combined with sensory evaluation, amino acid nitrogen, organic acids, and volatile compounds. This would make quality evaluation more traceable.

The findings may also support formula design with salt substitutes. Potassium chloride and other non-sodium salts are often used to partially replace NaCl. Yet excessive replacement may introduce bitter or metallic notes. Reviews on salt taste-enhancing peptides suggest that food-derived peptides may help improve saltiness perception and reduce dependence on sodium salts. Therefore, a combined strategy may be more realistic. Moderate NaCl reduction can be paired with partial KCl substitution and targeted enrichment of saltiness synergistic peptides. This approach may reduce sodium intake while preserving the typical salty and umami profile of doubanjiang.

The application should still be treated with caution. The present study confirms the effects of representative peptides under controlled addition conditions. It does not mean that these peptides alone can replace the full role of salt in fermentation. Salt still protects product safety and helps shape the fermentation ecology. The practical target should be balance. Enough salt is needed to preserve microbial stability and product character. Excessive sodium should be avoided because of health concerns. Peptide-guided regulation provides a middle path. It can help reduce salt pressure without flattening the flavor or weakening the traditional character of doubanjiang.

Overall, the findings can be applied in three linked directions. They can support the design of salt-reduced products. They can provide molecular markers for fermentation control. They can also guide the development of natural umami and saltiness-enhancing ingredients from fermented bean systems. This gives the study practical value beyond peptide identification. It moves doubanjiang improvement from empirical flavor adjustment toward evidence-based and matrix-specific taste regulation.

## 5. Conclusions

This study identified endogenous taste-active peptides in traditional doubanjiang and clarified their potential role in salt-reduction-oriented flavor regulation. A total of 1230 peptides were identified, among which 161 were predicted as potential umami peptides and 141 could enter the T1R1/T1R3 binding site in docking analysis. EESP, SCPH, SSSGF, PDTE, SYH, and DYDS were selected as representative candidates based on their docking performance and structural diversity. Their receptor recognition was mainly associated with polar anchoring, hydrogen bonding, electrostatic coordination, and hydrophobic fitting within the VFT domain. MM-GBSA analysis further indicated that favorable binding was not determined by acidic residues alone, but by the balance among polarity, hydrophobic matching, and conformational adaptation. Sensory validation showed that these peptides did not uniformly enhance umami in the doubanjiang matrix. Instead, all six peptides significantly increased saltiness, while PDTE showed the clearest umami-enhancing tendency and SSSGF, SYH, and SCPH showed stronger saltiness synergy. These findings suggest that umami peptides in complex fermented condiments may act as matrix-dependent taste modulators rather than simple umami enhancers. Overall, EESP, SCPH, SSSGF, PDTE, SYH, and DYDS can be regarded as representative endogenous umami-related peptides in traditional doubanjiang, with potential value for maintaining taste fullness during salt reduction. Future work should verify their formation, stability, and dose-dependent effects in real salt-reduced fermentation systems.

## Figures and Tables

**Table 1:** Specific scoring criteria for umami and saltiness of doubanjiang.

Score	Intensity Level	Umami Evaluation Criteria	Saltiness Evaluation Criteria
0	None	Umami is completely imperceptible	Saltiness is completely imperceptible
1	Extremely weak	Only faintly perceptible, with almost no clear umami impression	Only faintly perceptible, with almost no clear saltiness impression
2	Very weak	Umami is weak and disappears rapidly after tasting	Saltiness is weak and provides very limited support
3	Weak	Perceptible, but the taste body is thin and the duration is short	Perceptible, but weak and insufficient to support the overall flavor
4	Slightly weak	Umami is slightly perceptible, but lacks fullness	Saltiness is slightly perceptible, but the profile is not clear enough
5	Moderate	Umami is clear, with moderate intensity and a certain degree of thickness	Saltiness is clear, with moderate intensity and basic coordination
6	Moderately strong	Umami is relatively prominent, with good oral spreading and a relatively persistent aftertaste	Saltiness is relatively prominent, but still fairly well coordinated, without obvious irritation
7	Strong	Umami is obvious and full-bodied, with a relatively long aftertaste and good coordination with the overall flavor	Saltiness is obvious, with strong supporting effect and a relatively rounded overall impression
8	Very strong	Umami is highly prominent, with a thick taste body and strong persistence	Saltiness is very strong, but still basically clean, and can jointly produce a strong taste impact with umami
9	Extremely strong	Umami is extremely strong, very intense and persistent, and may be close to saturation	Saltiness is extremely strong, very intense, and already close to or at the saturation level

**Table 2:** Qualitative identification of peptides in doubanjiang and prediction of their potential umami contribution.

Numbers	Peptide	−10LgP	Mass	Length	*m*/*z*	RT	Area	PTM	Probability
1	AATAGV	27.42	488.26	6	489.27	6.38	22,700.00		0.969
2	ACFGV	25.29	495.22	5	496.22	4.48	10,200.00		0.953
3	AESH	29.92	442.18	4	443.19	2.24	14,000.00		0.923
4	ALVY	26.81	464.26	4	465.27	7.22	63,400.00		0.944
5	ARAC	30.99	419.20	4	420.20	7.52	88,900.00		0.917
6	ASCK	32.29	407.18	4	408.19	6.35	163,000.00		0.926
7	ASSFH	22.61	547.24	5	548.25	2.85	7620.00		0.948
8	CAGW	27.01	435.16	4	436.16	1.93	10,300.00		0.934
9	CHAM	24.31	460.16	4	461.17	2.48	33,200.00		0.934
10	CHW	28.44	444.16	3	445.16	4.81	233,000.00		0.933
11	CTGW	31.47	465.17	4	466.18	3.20	72,900.00		0.964
12	CTSS	37.54	396.13	4	397.14	3.86	80,700.00		0.942
13	CTTS	33.68	410.15	4	411.16	4.50	98,700.00		0.936
14	DEEH	32.17	528.18	4	529.19	3.50	16,300.00		0.932
15	DFD	26.76	395.13	3	396.14	3.52	10,700.00		0.908
16	DQAA	32.99	403.17	4	404.18	3.95	149,000.00		0.934
17	DSAGD	23.49	463.16	5	464.16	3.43	4290.00		0.922
18	DSSH	39.10	444.16	4	445.17	2.69	607,000.00		0.967
19	DTCK	34.13	465.19	4	466.20	6.03	42,000.00		0.911
20	DTSSS	40.10	495.18	5	496.20	3.85	181,000.00		0.937
21	DTYF	39.61	544.22	4	545.23	5.04	39,900.00		0.919
22	DYDS	27.29	498.16	4	499.17	4.37	25,300.00		0.932
23	EADA	33.71	404.15	4	405.16	6.77	13,500.00		0.927
24	ECPP	35.00	444.17	4	445.17	3.88	9870.00		0.923
25	EER	33.55	432.20	3	433.21	4.65	26,600.00		0.978
26	EESF	37.25	510.20	4	511.21	7.43	50,200.00		0.945
27	EEVG	38.79	432.19	4	433.20	6.85	120,000.00		0.905
28	EFD	40.92	409.15	3	410.16	4.26	62,200.00		0.99
29	EFGM	26.77	482.18	4	483.19	4.11	69,900.00		0.943
30	EFH	37.52	431.18	3	432.19	2.33	44,700.00		0.964
31	EHP	40.46	381.16	3	382.17	4.87	258,000.00		0.978
32	ELDE	41.70	504.21	4	505.22	3.97	192,000.00		0.925
33	ELEPFNLR	33.93	1016.53	8	509.27	4.13	121,000.00		0.985
34	EMGF	35.84	482.18	4	483.19	4.33	18,300.00		0.947
35	ENEN	34.85	504.18	4	505.19	4.42	8310.00		0.973
36	EPH	38.83	381.16	3	382.17	3.20	57,500.00		0.981
37	EPMY	32.15	538.21	4	539.21	2.42	7490.00		0.941
38	EQNEGKSVLSGFSAE	36.56	1580.73	15	791.38	3.67	24,800.00		0.974
39	ESKTV	22.60	562.30	5	563.30	7.65	33,800.00		0.918
40	ESY	37.45	397.15	3	398.16	2.75	5320.00		0.976
41	EVDQ	38.60	489.21	4	490.21	4.41	161,000.00		0.95
42	EVF	40.03	393.19	3	394.20	4.57	141,000.00		0.93
43	EVST	22.61	434.20	4	435.21	5.76	58,900.00		0.97
44	EVYE	30.13	538.23	4	539.24	3.47	6180.00		0.906
45	FCVS	33.76	454.19	4	455.20	4.21	156,000.00		0.929
46	FDLPL	39.03	603.33	5	604.34	5.97	28,300.00		0.907
47	FGHD	33.81	474.19	4	475.20	2.53	15,100.00		0.943
48	FGTTS	25.72	511.23	5	512.24	4.32	62,000.00		0.955
49	FLED	36.11	522.23	4	523.24	3.03	586,000.00		0.915
50	FSEE	36.77	510.20	4	511.21	2.83	34,200.00		0.941
51	FSR	39.83	408.21	3	409.22	5.55	35,000.00		0.985
52	FTM	40.58	397.17	3	398.17	6.53	25,700.00		0.931
53	FYIGGNPEAEFPETQE	43.51	1826.80	16	914.41	5.10	548,000.00		0.977
54	GCSSS	22.61	439.14	5	440.15	4.90	18,800.00		0.92
55	GGDCR	25.28	506.19	5	507.20	4.89	54,500.00		0.919
56	GNNIFSGFKRD	37.73	1253.62	11	418.88	3.26	117,000.00		0.967
57	GVAEF	23.08	521.25	5	522.26	4.49	8220.00		0.908
58	GVSSELEPFNLR	42.65	1346.68	12	674.35	4.40	221,000.00		0.928
59	HAET	25.46	456.20	4	457.21	4.15	20,400.00		0.911
60	HSCF	26.83	492.18	4	493.18	4.97	110,000.00		0.929
61	HSCL	31.95	458.19	4	459.20	4.37	113,000.00		0.939
62	HSDE	31.27	486.17	4	487.18	2.27	14,500.00		0.943
63	ISLTDTGSSNNQLDQMPRRFY	26.84	2458.15	21	820.40	3.46	81,800.00	Oxidation (M)	0.91
64	KAVC	24.31	419.22	4	420.23	6.13	16,900.00		0.935
65	KCF	36.98	396.18	3	397.19	5.28	10,500.00		0.926
66	KKGEA	25.70	531.30	5	532.31	7.03	154,000.00		0.967
67	KNY	36.23	423.21	3	424.22	6.70	6050.00		0.941
68	LDTSNIANQL	40.47	1087.55	10	544.79	4.29	506,000.00		0.945
69	LDTSNIANQLDSTPRVF	43.51	1889.95	17	945.99	5.04	1,190,000.00		0.937
70	LDTSNTLNQLDSTPRLF	43.51	1933.97	17	968.00	5.36	36,000.00		0.939
71	LDTSNTLNQLDSTPRVF	43.50	1919.96	17	960.99	4.82	20,400.00		0.927
72	LEMY	34.11	570.24	4	571.24	4.09	76,300.00	Oxidation (M)	0.939
73	LGGNPEVEFPET	22.61	1287.60	12	644.81	4.35	23,200.00		0.967
74	LLDTSNIANQLDSTPRVF	43.52	2003.03	18	668.69	5.45	255,000.00		0.947
75	LLDTSNTLNQLDSTPRL	43.53	1899.99	17	951.01	4.76	23,100.00		0.934
76	MACF	32.33	470.17	4	471.18	4.87	126,000.00		0.928
77	MCVH	24.39	488.19	4	489.20	2.59	165,000.00		0.98
78	MDDPP	23.68	573.21	5	574.22	4.86	4330.00		0.928
79	MGML	24.44	466.19	4	467.21	2.49	51,800.00	Oxidation (M)	0.914
80	MGYN	26.76	483.18	4	484.19	2.40	25,600.00		0.902
81	MKC	34.97	380.16	3	381.17	4.28	972,000.00		0.955
82	MKCC	28.30	483.16	4	484.17	4.22	195,000.00		0.928
83	MMTE	24.31	526.18	4	527.19	2.61	500,000.00	Oxidation (M)	0.967
84	MPSM	26.80	480.17	4	481.19	5.08	301,000.00	Oxidation (M)	0.933
85	MRF	24.57	452.22	3	453.23	6.18	33,700.00		0.959
86	MRS	35.64	392.18	3	393.19	5.59	14,100.00		0.983
87	MSAT	25.70	424.16	4	425.17	5.17	76,700.00	Oxidation (M)	0.96
88	MSF	35.43	399.15	3	400.15	4.31	99,100.00	Oxidation (M)	0.959
89	MSR	41.47	392.18	3	393.20	2.15	6000.00		0.977
90	MSTS	28.01	424.16	4	425.17	5.83	39,200.00		0.925
91	MTGE	38.09	436.16	4	437.17	3.10	254,000.00		0.908
92	MVD	36.74	379.14	3	380.15	3.15	389,000.00	Oxidation (M)	0.954
93	MVME	39.52	508.20	4	509.22	4.17	123,000.00		0.988
94	MVSC	40.24	438.16	4	439.17	4.04	339,000.00		0.938
95	MVYA	31.03	498.21	4	499.22	2.49	43,300.00	Oxidation (M)	0.947
96	MYSG	23.08	472.16	4	473.17	6.30	411,000.00	Oxidation (M)	0.971
97	MYT	41.69	413.16	3	414.17	3.35	207,000.00		0.948
98	NANL	24.56	430.22	4	431.22	3.66	27,400.00		0.929
99	NEPE	24.91	487.19	4	488.21	2.63	158,000.00		0.95
100	NEY	40.66	424.16	3	425.17	6.09	55,100.00		0.959
101	NME	38.00	392.14	3	393.14	4.51	1,350,000.00		0.989
102	NMSF	25.32	513.19	4	514.20	2.55	122,000.00	Oxidation (M)	0.957
103	NQLDSTPRVF	24.68	1175.59	10	588.81	3.58	166,000.00		0.928
104	NSME	27.00	479.17	4	480.17	2.95	60,100.00		0.981
105	NTCK	26.80	464.21	4	465.22	6.05	8890.00		0.943
106	NYEE	28.42	553.20	4	554.22	4.84	30,300.00		0.96
107	PDSH	23.08	454.18	4	455.19	2.22	14,700.00		0.908
108	PDTE	36.82	460.18	4	461.19	2.89	372,000.00		0.975
109	PEGH	36.27	438.19	4	439.19	2.73	10,500.00		0.915
110	PHE	37.50	381.16	3	382.17	3.84	34,700.00		0.979
111	PSEE	32.94	460.18	4	461.19	4.84	280,000.00		0.941
112	QCR	28.28	405.18	3	406.19	1.99	8780.00		0.967
113	QDEL	33.91	503.22	4	504.24	2.78	27,600.00		0.94
114	RAVCE	22.25	576.27	5	577.28	7.18	61,200.00		0.915
115	RCQ	31.07	405.18	3	406.19	5.15	23,600.00		0.961
116	RDGS	35.53	433.19	4	434.20	4.38	137,000.00		0.947
117	SCPH	30.25	442.16	4	443.17	2.04	9480.00		0.966
118	SEEQNEGKSVLSGFSAE	43.49	1796.81	17	899.42	3.69	190,000.00		0.938
119	SEEQNKGKSVLSGFSAE	31.58	1795.86	17	599.63	2.99	181,000.00		0.94
120	SEGM	26.83	438.14	4	439.15	5.37	52,200.00	Oxidation (M)	0.951
121	SPEE	40.90	460.18	4	461.19	3.30	233,000.00		0.943
122	SRM	38.00	392.18	3	393.19	7.24	156,000.00		0.975
123	SSMA	35.82	410.15	4	411.16	4.80	111,000.00	Oxidation (M)	0.956
124	SSSGF	22.35	483.20	5	484.21	6.14	75,100.00		0.91
125	SVKT	22.61	433.25	4	434.26	7.40	65,000.00		0.928
126	SYH	40.82	405.16	3	406.17	2.27	22,600.00		0.975
127	TCTS	24.31	410.15	4	411.16	6.14	39,100.00		0.936
128	TCTT	24.31	424.16	4	425.17	4.45	33,100.00		0.912
129	TEEP	37.77	474.20	4	475.21	4.71	52,000.00		0.934
130	TEEPP	26.75	571.25	5	572.26	5.03	220,000.00		0.933
131	THSF	23.08	490.22	4	491.23	6.78	5810.00		0.984
132	TMF	32.18	413.16	3	414.17	4.00	142,000.00	Oxidation (M)	0.921
133	TSNIANQLDSTPRVF	43.23	1661.84	15	831.93	4.30	44,400.00		0.903
134	TSSM	32.71	440.16	4	441.17	3.96	75,400.00	Oxidation (M)	0.921
135	TTCR	37.75	479.22	4	480.22	2.32	163,000.00		0.922
136	TTSF	32.40	454.21	4	455.22	2.09	653,000.00		0.924
137	VCW	26.86	406.17	3	407.18	7.03	15,800.00		0.965
138	VFD	42.60	379.17	3	380.18	2.61	476,000.00		0.974
139	VMY	33.65	427.18	3	428.18	3.15	395,000.00	Oxidation (M)	0.907
140	VPTF	42.76	462.25	4	463.26	3.65	32,900.00		0.977
141	VSDH	39.65	456.20	4	457.21	4.02	18,200.00		0.977
142	VSSGC	24.47	451.17	5	452.18	1.85	5520.00		0.91
143	VSTE	25.92	434.20	4	435.21	6.84	69,400.00		0.973
144	VTCD	31.61	436.16	4	437.17	2.44	13,800.00		0.918
145	YCV	37.92	383.15	3	384.16	4.86	223,000.00		0.964
146	YDLY	24.31	572.25	4	573.26	3.04	43,800.00		0.978
147	YLGGNPEVE	32.75	976.45	9	489.24	3.04	116,000.00		0.988
148	YLGGNPEVEFPE	30.53	1349.61	12	675.82	4.83	592,000.00		0.986
149	YLGGNPEVEFPET	39.29	1450.66	13	726.34	4.75	252,000.00		0.991
150	YMA	42.11	383.15	3	384.16	3.52	586,000.00		0.941
151	YMTA	25.28	500.19	4	501.20	2.09	9100.00	Oxidation (M)	0.905
152	YMV	36.89	427.18	3	428.18	2.56	1,190,000.00	Oxidation (M)	0.907
153	YNGN	28.92	466.18	4	467.19	2.60	13,300.00		0.984
154	YRK	26.75	465.27	3	466.28	7.61	11,100.00		0.939
155	YSET	30.98	498.20	4	499.21	5.48	48,800.00		0.921
156	YSM	40.86	399.15	3	400.15	5.68	191,000.00		0.912
157	YTH	26.74	419.18	3	420.19	3.76	40,000.00		0.963
158	YTM	35.20	413.16	3	414.17	5.17	117,000.00		0.947
159	YTSG	36.61	426.18	4	427.19	5.50	97,200.00		0.964
160	YTT	40.41	383.17	3	384.18	3.90	136,000.00		0.944
161	YVM	40.51	411.18	3	412.19	5.12	112,000.00		0.906

Note: Peptide indicates the peptide sequence. −10LgP indicates the PEAKS identification score. A higher value indicates higher confidence in identification, and in the PEAKS system this value is converted from the *p*-value. Mass indicates the monoisotopic molecular mass of the peptide. Length indicates the number of amino acid residues in the peptide. *m*/*z* indicates the mass-to-charge ratio. RT indicates the retention time. Area indicates the chromatographic peak area of the peptide. PTM indicates the post-translational modification information detected for the peptide. Probability indicates the predicted probability from UMPred-FRL that the peptide is a potential umami peptide. The wording for −10LgP follows PEAKS usage, and the description of Probability follows the UMPred-FRL predictor’s output convention.

**Table 3:** Docking binding energies of 141 umami peptides with the T1R1/T1R3 receptor.

Peptide	Docking Score	Glide Ecoul	Glide Emodel	Glide Energy	Glide Evdw	Glide Gscore	Glide Hbond
EESP	−7.56	−24.54	−84.91	−63.11	−38.57	−7.56	−1.46
SCPH	−7.30	−19.14	−80.33	−58.84	−39.70	−8.14	−2.14
SSSGF	−7.03	−23.77	−77.79	−63.16	−39.39	−7.39	−1.64
PDTE	−6.85	−26.71	−79.71	−55.39	−28.68	−6.85	−1.20
SYH	−6.81	−24.51	−92.53	−60.48	−35.97	−7.70	−1.25
DYDS	−6.74	−24.49	−81.20	−59.54	−35.05	−6.74	−1.18
HAET	−6.69	−24.93	−79.27	−59.35	−34.42	−7.62	−1.64
SRM	−6.63	−25.81	−83.64	−49.87	−24.07	−7.05	−1.61
KKGEA	−6.53	−27.90	−94.45	−64.55	−36.65	−6.77	−1.15
NANL	−6.53	−17.51	−73.62	−54.76	−37.25	−6.70	−1.42
EADA	−6.52	−20.78	−70.71	−50.48	−29.70	−6.52	−0.97
VSSGC	−6.46	−21.66	−79.39	−54.05	−32.39	−6.66	−1.61
FSR	−6.42	−24.45	−69.29	−48.48	−24.03	−6.70	−1.27
DTCK	−6.41	−22.73	−84.42	−59.57	−36.84	−6.58	−0.50
CHAM	−6.39	−15.72	−77.35	−58.65	−42.92	−6.74	−1.54
YNGN	−6.39	−21.54	−88.82	−63.09	−41.54	−6.97	−1.44
AESH	−6.31	−21.15	−79.40	−56.94	−35.79	−6.86	−1.10
ASCK	−6.27	−18.10	−76.55	−49.56	−31.46	−6.41	−0.62
PDSH	−6.26	−21.96	−81.24	−58.03	−36.07	−6.80	−1.05
MRS	−6.23	−27.27	−81.17	−53.85	−26.58	−6.62	−0.92
DTYF	−6.23	−10.11	−77.18	−58.73	−48.62	−6.39	−0.56
ELDE	−6.22	−20.79	−76.70	−60.84	−40.05	−6.36	−0.80
HSDE	−6.22	−21.58	−80.97	−57.20	−35.63	−6.29	−0.95
VSTE	−6.22	−24.23	−74.65	−55.64	−31.41	−6.41	−0.93
YSET	−6.12	−19.75	−76.93	−55.94	−36.19	−6.70	−0.87
RCQ	−6.11	−20.51	−79.98	−51.90	−31.39	−6.40	−1.47
VSDH	−6.09	−23.88	−84.94	−59.63	−35.75	−6.81	−0.93
ACFGV	−6.05	−19.58	−84.65	−64.73	−45.15	−6.20	−0.62
YMTA	−6.04	−18.96	−74.56	−58.50	−39.54	−6.37	−0.72
ARAC	−6.02	−22.15	−74.16	−51.08	−28.93	−6.15	−1.06
EFH	−6.00	−15.61	−73.83	−54.47	−38.86	−6.73	−0.50
NTCK	−5.98	−16.30	−78.46	−58.70	−42.40	−6.12	−0.58
ESKTV	−5.93	−18.81	−83.36	−60.47	−41.66	−6.45	−0.83
DTSSS	−5.92	−29.26	−77.18	−61.29	−32.03	−6.76	−1.95
NME	−5.92	−21.26	−67.40	−50.97	−29.71	−6.09	−1.13
MVME	−5.91	−19.53	−78.79	−57.64	−38.11	−6.34	−1.18
NSME	−5.90	−28.17	−88.21	−64.57	−36.40	−6.82	−2.17
MMTE	−5.87	−18.99	−74.05	−57.13	−38.14	−6.27	−0.84
MKC	−5.85	−21.62	−69.59	−48.01	−26.38	−6.28	−1.49
TCTT	−5.85	−19.91	−61.75	−54.10	−34.19	−6.36	−1.69
SEGM	−5.83	−23.34	−74.58	−55.91	−32.57	−6.25	−0.95
TMF	−5.81	−20.28	−65.58	−47.16	−26.88	−6.09	−0.37
NEY	−5.75	−17.53	−69.14	−53.98	−36.45	−5.93	−0.70
EEVG	−5.74	−21.92	−69.53	−53.55	−31.62	−5.74	−0.75
QDEL	−5.67	−14.42	−65.36	−53.17	−38.75	−5.67	−0.79
YDLY	−5.66	−14.52	−78.95	−57.80	−43.28	−6.24	−0.40
HSCF	−5.65	−16.14	−74.52	−56.09	−39.95	−6.08	−1.44
TEEP	−5.65	−14.30	−68.07	−46.96	−32.65	−5.65	−0.92
EFD	−5.65	−16.92	−63.58	−48.19	−31.26	−5.65	−0.65
MTGE	−5.64	−20.08	−72.05	−49.54	−29.46	−6.09	−1.41
ASSFH	−5.63	−18.51	−85.35	−64.66	−46.15	−6.27	−0.78
ECPP	−5.60	−12.15	−58.54	−43.08	−30.93	−6.13	−1.24
KCF	−5.56	−21.38	−71.33	−48.70	−27.32	−5.85	−0.82
EMGF	−5.56	−13.94	−67.63	−53.74	−39.80	−6.11	−1.55
NMSF	−5.54	−17.72	−72.12	−59.05	−41.33	−5.72	−1.58
DEEH	−5.53	−18.13	−70.17	−53.14	−35.01	−5.54	−0.21
MYT	−5.53	−17.69	−69.99	−51.97	−34.28	−5.97	−0.52
RAVCE	−5.50	−23.65	−83.56	−62.24	−38.59	−6.10	−0.80
SVKT	−5.50	−21.83	−71.60	−56.66	−34.83	−5.89	−0.92
FGHD	−5.49	−17.15	−68.32	−58.35	−41.20	−6.05	−0.96
EER	−5.46	−21.97	−63.26	−43.37	−21.40	−5.76	−0.96
VCW	−5.46	−12.49	−62.98	−47.66	−35.16	−5.69	−0.59
NYEE	−5.46	−19.16	−83.03	−62.86	−43.70	−7.99	−1.95
MYSG	−5.45	−22.60	−76.19	−52.83	−30.23	−5.90	−1.13
KAVC	−5.45	−18.61	−68.08	−50.84	−32.24	−6.11	−1.22
THSF	−5.42	−20.01	−79.79	−57.98	−37.96	−6.42	−1.17
TEEPP	−5.42	−11.41	−69.45	−51.94	−40.52	−5.42	−0.17
ENEN	−5.41	−15.85	−72.95	−57.01	−41.16	−5.41	−0.74
QCR	−5.39	−21.06	−69.27	−47.05	−26.00	−5.78	−0.53
EVDQ	−5.38	−12.63	−63.32	−50.13	−37.50	−5.38	−0.83
TTSF	−5.30	−15.80	−67.42	−51.29	−35.49	−5.62	−0.83
CHW	−5.28	−12.60	−68.20	−54.81	−42.20	−5.63	−0.73
MKCC	−5.28	−20.55	−79.29	−61.54	−40.99	−5.71	−1.37
YTSG	−5.27	−19.73	−69.96	−50.37	−30.63	−5.85	−0.91
VMY	−5.27	−17.02	−67.01	−48.06	−31.04	−5.44	−0.66
EVST	−5.27	−16.67	−64.88	−51.82	−35.16	−5.81	−1.10
RDGS	−5.25	−22.32	−71.28	−49.94	−27.62	−5.82	−1.58
MVD	−5.24	−20.77	−58.45	−44.09	−23.32	−5.63	−0.92
YTH	−5.22	−19.62	−68.01	−52.94	−33.32	−6.03	−0.70
DFD	−5.15	−13.33	−55.98	−39.48	−26.16	−5.15	−0.91
FTM	−5.14	−9.67	−60.11	−48.24	−38.57	−5.42	−0.68
ESY	−5.09	−16.36	−60.79	−44.99	−28.63	−5.41	−0.73
YMA	−5.07	−16.38	−66.07	−48.05	−31.67	−5.61	−0.74
NEPE	−5.04	−15.24	−72.92	−56.15	−40.91	−5.05	−0.33
MPSM	−5.03	−15.22	−66.59	−51.75	−36.53	−5.24	−0.66
CTSS	−5.01	−14.71	−53.23	−44.07	−29.36	−5.13	−1.23
PEGH	−5.00	−27.52	−77.93	−54.01	−26.49	−6.39	−1.05
FGTTS	−4.99	−17.25	−73.22	−62.11	−44.86	−5.28	−1.59
LEMY	−4.98	−11.60	−66.98	−51.61	−40.01	−5.18	−0.60
EVF	−4.97	−14.51	−54.02	−44.23	−29.72	−5.28	−0.44
EPH	−4.97	−18.75	−64.44	−46.14	−27.39	−5.66	−0.48
YCV	−4.97	−21.28	−62.16	−46.25	−24.97	−5.60	−0.47
MDDPP	−4.95	−5.52	−64.88	−52.01	−46.49	−4.95	−0.32
TCTS	−4.93	−20.70	−67.30	−48.80	−28.10	−5.26	−1.04
VFD	−4.91	−15.91	−56.45	−46.25	−30.33	−5.68	−0.91
YTT	−4.90	−13.50	−49.64	−41.07	−27.57	−5.18	−0.96
EHP	−4.87	−20.57	−63.19	−47.67	−27.10	−5.45	−0.50
MRF	−4.87	−18.63	−73.59	−50.71	−32.08	−5.27	−0.37
YMV	−4.85	−14.90	−63.23	−45.09	−30.19	−5.40	−0.87
CTTS	−4.85	−17.87	−56.09	−47.85	−29.98	−4.97	−1.26
MSF	−4.85	−16.14	−64.95	−47.81	−31.67	−5.28	−0.47
EVYE	−4.84	−10.41	−59.68	−48.86	−38.45	−4.85	−0.64
GGDCR	−4.84	−19.47	−73.47	−55.05	−35.58	−5.73	−0.84
HSCL	−4.84	−20.35	−67.77	−52.05	−31.70	−5.27	−0.72
TSSM	−4.84	−16.84	−65.85	−49.42	−32.58	−5.15	−0.92
CTGW	−4.80	−21.20	−73.12	−56.51	−35.30	−5.83	−0.78
GCSSS	−4.78	−23.54	−67.49	−52.08	−28.54	−4.94	−0.97
YRK	−4.76	−23.62	−76.65	−58.02	−34.39	−5.07	−1.15
SSMA	−4.75	−20.50	−67.01	−50.04	−29.54	−5.25	−0.53
CAGW	−4.73	−9.51	−58.22	−46.57	−37.07	−4.73	−0.38
DSAGD	−4.71	−17.60	−64.53	−49.99	−32.39	−4.71	−0.47
ALVY	−4.70	−17.71	−62.21	−47.65	−29.93	−4.84	−0.65
MVSC	−4.69	−18.20	−57.63	−49.56	−31.36	−5.14	−1.01
MGYN	−4.66	−10.74	−69.17	−52.42	−41.68	−5.09	−0.73
FSEE	−4.63	−9.14	−59.33	−45.97	−36.83	−4.63	−0.42
YTM	−4.61	−15.02	−57.31	−48.52	−33.50	−4.89	−0.49
FLED	−4.60	−12.63	−58.89	−49.91	−37.27	−4.60	−0.29
EPMY	−4.59	−18.54	−70.18	−59.08	−40.54	−4.76	−0.42
DQAA	−4.58	−16.74	−61.38	−51.10	−34.36	−5.49	−0.87
MVYA	−4.57	−11.90	−69.54	−52.12	−40.22	−4.98	−0.72
MSR	−4.55	−19.75	−60.62	−44.91	−25.16	−4.99	−0.94
DSSH	−4.53	−18.90	−63.97	−48.30	−29.41	−5.79	−1.51
KNY	−4.50	−16.81	−66.65	−49.38	−32.57	−5.14	−0.70
MSAT	−4.47	−17.39	−62.00	−44.35	−26.96	−4.92	−1.28
EFGM	−4.45	−8.52	−62.98	−50.09	−41.57	−4.97	−0.70
YSM	−4.37	−13.08	−52.35	−45.41	−32.33	−4.65	−0.47
YVM	−4.35	−12.40	−56.02	−48.86	−36.46	−4.67	−1.33
PHE	−4.32	−25.15	−69.24	−47.52	−22.38	−6.42	−0.60
FCVS	−4.32	−9.94	−57.45	−48.65	−38.71	−4.72	−0.88
MCVH	−4.32	−16.94	−70.05	−56.54	−39.60	−5.26	−0.48
MSTS	−4.29	−15.23	−59.56	−45.43	−30.20	−4.73	−1.06
GVAEF	−4.24	−11.74	−64.40	−54.49	−42.75	−5.20	−0.57
MGML	−4.22	−13.92	−68.71	−51.65	−37.74	−4.65	−0.02
VPTF	−4.20	−9.98	−57.40	−46.80	−36.82	−4.27	−0.61
AATAGV	−4.10	−12.44	−64.80	−52.88	−40.44	−4.23	−0.35
SPEE	−4.05	−7.81	−49.50	−40.79	−32.97	−4.05	−0.67
MACF	−3.81	−10.64	−61.12	−49.38	−38.75	−4.28	−0.16
VTCD	−3.64	−18.97	−53.52	−45.73	−26.76	−3.84	−1.03
TTCR	−3.46	−18.64	−64.71	−53.51	−34.87	−3.78	−0.80
FDLPL	−3.09	−2.59	−48.05	−43.42	−40.83	−3.67	−0.02
PSEE	−2.86	−14.24	−55.49	−46.52	−32.28	−2.86	−0.78

Note: All energy terms in the table are expressed in kcal/mol. Peptide indicates the umami peptide sequence. Docking score indicates the overall molecular docking score, and a lower value generally indicates a stronger binding tendency between the ligand and the receptor. Glide Ecoul indicates the Coulomb electrostatic interaction energy between the ligand and the receptor. Glide Evdw indicates the van der Waals interaction energy between the ligand and the receptor. Glide energy is the sum of Glide Ecoul and Glide Evdw, and is used to characterize the non-bonded interaction energy. Glide Emodel is a composite energy term used for pose selection. It is mainly composed of Coulomb energy, van der Waals energy, and Glide Gscore, and is commonly used to identify the preferred binding pose of the same ligand. Glide Gscore indicates the score given by the Glide empirical scoring function and is used to evaluate the overall binding performance between the ligand and the receptor. Glide Hbond indicates the contribution of hydrogen bonding to the docking score.

**Table 4:** MM-GBSA binding energies of six umami peptides with their receptor.

Peptide	MMGBSA dG Bind (NS)	MMGBSA dG Bind (NS) Coulomb	MMGBSA dG Bind (NS) Hbond	MMGBSA dG Bind (NS) Lipo	MMGBSA dG Bind (NS) Packing	MMGBSA dG Bind (NS) Solv GB	MMGBSA dG Bind (NS) vdW
EESP	−46.118	252.443	−5.658	−11.219	0.000	−221.768	−59.915
SCPH	−81.214	32.086	−5.508	−11.998	−1.987	−45.905	−47.902
SSSGF	−73.338	−0.137	−5.951	−12.287	−1.154	−6.651	−47.158
PDTE	−37.295	265.905	−7.146	−8.472	0.000	−238.647	−48.934
SYH	−79.991	12.043	−5.164	−11.957	−1.337	−30.390	−43.187
DYDS	−49.387	242.164	−6.236	−9.702	−0.001	−220.754	−54.858

Note: All energy terms in the table are expressed in kcal/mol. MMGBSA dG Bind (NS) indicates the no-strain binding free energy, where NS stands for no strain, meaning that the strain energy arising from conformational changes in the receptor and ligand during complex formation is not included. MMGBSA dG Bind (NS) Coulomb indicates the contribution of Coulomb electrostatic interactions. MMGBSA dG Bind (NS) Hbond indicates the hydrogen-bond contribution. MMGBSA dG Bind (NS) Lipo indicates the lipophilic or hydrophobic interaction contribution. MMGBSA dG Bind (NS) Packing indicates the packing correction term, which mainly reflects contributions from π–π stacking and local close packing. MMGBSA dG Bind (NS) Solv GB indicates the contribution of polar solvation energy calculated based on the Generalized Born model. MMGBSA dG Bind (NS) vdW indicates the contribution of van der Waals interactions. In general, a more negative value indicates a greater contribution of that term to complex stabilization.

**Table 5:** Basic information, taste descriptions, and thresholds of the six screened umami peptides.

Peptide	Mass	Taste Description	Umami Threshold (mmol/L)
EESP	510.196	A relatively distinct umami taste, accompanied by a slight sour note and an extremely weak bitter-astringent aftertaste.	0.49
SCPH	442.164	A mild and relatively full-bodied umami taste, possibly accompanied by a subtle drying sensation.	0.141
SSSGF	483.197	A relatively mild umami profile, with a soft onset and a slight bitter note in the later stage.	0.259
PDTE	460.181	A relatively clear umami taste with certain persistence, accompanied by a slight sour-umami note.	1.087
SYH	405.165	A relatively rounded umami profile, with an overall smooth mouthfeel, although a slight bitterness may appear at the end.	0.154
DYDS	498.16	A relatively distinct sour-umami composite sensation, with high umami recognizability, accompanied by a slight bitter aftertaste and a certain lingering sensation.	0.502

## Data Availability

The original contributions presented in this study are included in the article/supplementary material. Further inquiries can be directed to the corresponding author.

## Supplementary Materials

The following supporting information can be downloaded at: https://www.mdpi.com/article/10.3390/foods15101819/s1, Supplementary S1: Polypeptide FASTA Format Converter; Supplementary S2: Qualitative identification of peptides; Table S1: Experimentally validated umami and umami-enhancing peptides reported in fermented foods.
