# Supplementary material for: Discovery and Validation of Novel Umami Peptides from Traditional Broad Bean Paste (Doubanjiang)
Source: Foods. 2026 May 21;15(10):1819. doi: 10.3390/foods15101819 (PMC13205413; doi:10.3390/foods15101819)
Supplement: Supplementary file 1 [file foods-15-01819-s001.zip › Table S1.pdf]

Supplementary Table S1. Experimentally validated umami and umami-enhancing peptides reported in fermented foods

Fermented food source	Confirmed umami or umami-enhancing peptides	Main validation evidence	Taste threshold or main taste effect	Class	Probability
Soy sauce	LPEEV	Isolation, synthesis, sensory evaluation	Umami threshold 0.43 mmol/L. It also showed umami-enhancing activity.	Umami	0.932
Soy sauce	ALPEEV	Isolation, synthesis, sensory evaluation	Weak umami taste, but clear umami-enhancing activity. Umami-enhancing threshold 1.52 mmol/L.	Umami	0.945
Fermented soybean curd, sufu	DPRVI	Peptidomics, synthesis, sensory evaluation	Reported as a taste-active umami or umami-enhancing peptide.	Umami	0.974
Fermented soybean curd, sufu	THEA	Peptidomics, synthesis, sensory evaluation	Reported as a taste-active umami or umami-enhancing peptide.	Umami	0.972
Fermented soybean curd, sufu	TD	Peptidomics, synthesis, sensory evaluation	Reported as a taste-active umami or umami-enhancing peptide.	Umami	0.949
Fermented soybean curd, sufu	GE	Peptidomics, synthesis, sensory evaluation	Reported as a taste-active umami or umami-enhancing peptide.	Umami	0.961
Fermented soybean curd, sufu	VD	Peptidomics, synthesis, sensory evaluation	Reported as a taste-active umami or umami-enhancing peptide.	Umami	0.972
Douchi	EALEATAQ	Bioinformatics screening, sensory threshold, T1R1/T1R3 mechanism analysis	Umami threshold 0.139 mg/mL.	Umami	0.972
Douchi	SPPTEE	Bioinformatics screening, sensory threshold, T1R1/T1R3 mechanism analysis	Umami threshold 0.085 mg/mL.	Umami	0.948
Douchi	SEEG	Bioinformatics screening, sensory threshold, T1R1/T1R3 mechanism analysis	Umami threshold 0.096 mg/mL.	Umami	0.968
Douchi	KEE	Bioinformatics screening, sensory threshold, T1R1/T1R3 mechanism analysis	Umami threshold 0.060 mg/mL.	Umami	0.958

Fermented food source	Confirmed umami or umami-enhancing peptides	Main validation evidence	Taste threshold or main taste effect	Class	Probability
Douchi	FEE	Bioinformatics screening, sensory threshold, T1R1/T1R3 mechanism analysis	Umami threshold 0.079 mg/mL.	Umami	0.993
Douchi	VGSEL	Sensory threshold, molecular docking	Umami threshold within 0.5 to 2.0 mg/mL.	Umami	0.863
Douchi	VD	Sensory evaluation, threshold determination	Umami threshold 0.16 mg/mL.	Umami	0.972
Tauco	LVTGGDSGIGR	Peptidomics-sensomics, taste dilution analysis, HR-LC-MS/MS, molecular docking	Associated with intense umami taste in tauco fractions.	Umami	0.87
Tauco	DVTDVTGDRGVTVT	Peptidomics-sensomics, taste dilution analysis, HR-LC-MS/MS, molecular docking	One of the 3 peptides with the lowest docking energy to T1R1/T1R3, at -9.1 kcal/mol.	Umami	0.943
Tauco	LVNNDDRDSY	Peptidomics-sensomics, taste dilution analysis, HR-LC-MS/MS, molecular docking	Associated with intense umami taste in tauco fractions.	Umami	0.924
Fermented wheat gluten hydrolysates	QQLPQFEE	Machine learning screening, sensory evaluation, electronic tongue, molecular docking	Umami peptide with umami-enhancing or saltiness-enhancing effect. It had the lowest threshold among the reported candidates.	Umami	0.823
Fermented wheat gluten hydrolysates	EELR	Machine learning screening, sensory evaluation, electronic tongue, molecular docking	Umami peptide with umami-enhancing or saltiness-enhancing effect.	Umami	0.966
Fermented wheat gluten hydrolysates	YTCE	Machine learning screening, sensory evaluation, electronic tongue, molecular docking	Umami peptide with umami-enhancing or saltiness-enhancing effect.	Umami	0.806
Fermented wheat gluten hydrolysates	EEDQ	Machine learning screening, sensory evaluation, electronic tongue, molecular docking	It showed the highest umami intensity among the reported candidates.	Umami	0.94

Fermented food source	Confirmed umami or umami-enhancing peptides	Main validation evidence	Taste threshold or main taste effect	Class	Probability
Fermented wheat germ	EEL	Molecular docking, electronic tongue analysis	Reported as a novel umami peptide in fermented wheat germ.	Umami	0.899
Fermented wheat germ	VDSE	Molecular docking, electronic tongue analysis	Reported as a novel umami peptide in fermented wheat germ.	Umami	0.906
Huangjiu	TYNPR	Sensory-guided isolation, peptidomics, molecular docking, sensory validation	Umami and umami-enhancing peptide. Reported threshold 2.11 mmol/L.	Umami	0.984
Huangjiu	SYNPR	Sensory-guided isolation, peptidomics, molecular docking, sensory validation	Umami and umami-enhancing peptide.	Umami	0.949
Huangjiu	RFRQGD	Sensory-guided isolation, peptidomics, molecular docking, sensory validation	Identified as a novel umami peptide.	Umami	0.8
Huangjiu	NFHHGD	Sensory-guided isolation, peptidomics, molecular docking, sensory validation	Umami and umami-enhancing peptide.	Umami	0.866
Huangjiu	FHHGD	Sensory-guided isolation, peptidomics, molecular docking, sensory validation	Umami and umami-enhancing peptide.	Umami	0.935
Fermented grains, jiupei	EFFSNYGTRV	Metagenomics, machine learning, sensory validation, molecular docking	Umami-enhancing threshold within 0.09 to 0.116 mmol/L.	Umami	0.987
Fermented grains, jiupei	EFFSNYDTRL	Metagenomics, machine learning, sensory validation, molecular docking	It showed the best umami property among the reported candidates.	Umami	0.971
Fermented grains, jiupei	GCWGRGRLCQW	Metagenomics, machine learning, sensory validation, molecular docking	Umami-enhancing threshold within 0.09 to 0.116 mmol/L.	Umami	0.977
Fermented sea bass	FDD	Fractionation, RP-HPLC-MS, sensory evaluation, electronic tongue, molecular dynamics	Umami threshold within 0.0391 to 0.0938 mg/mL. Additive umami-enhancing effect.	Umami	0.901

Fermented food source	Confirmed umami or umami-enhancing peptides	Main validation evidence	Taste threshold or main taste effect	Class	Probability
Fermented sea bass	EDEI	Fractionation, RP-HPLC-MS, sensory evaluation, electronic tongue, molecular dynamics	Umami threshold within 0.0391 to 0.0938 mg/mL. Synergistic umami-enhancing effect.	Umami	0.895
Fermented goose bone broth	VGYDAE	Sequential chromatography, sensory analysis, nano-LC-MS/MS, synthesis	Umami-enhancing threshold within 0.41 to 1.15 mmol/L.	Umami	0.924
Chinese anchovy sauce	TREQMIHER	Same as above	Umami with slight sourness and bitterness. Threshold 0.25 mg/mL. Umami-enhancing threshold 0.5 mg/mL.	Umami	0.84
Chinese anchovy sauce	IMEALAGAGIDPRR	Same as above	Umami with slight bitterness and sourness. Threshold 0.2 mg/mL. Umami-enhancing threshold 0.2 mg/mL.	Umami	0.898
Chinese anchovy sauce	NQEGLFR	Same as above	Umami and bitterness with slight sourness. Threshold 0.2 mg/mL. Umami-enhancing threshold 0.2 mg/mL.	Umami	0.81
Manchego cheese	PSE	Sensory evaluation, MS identification	Identified as an umami peptide in Manchego cheese.	Umami	0.953
Manchego cheese	RKE	Sensory evaluation, MS identification	Identified as an umami peptide in Manchego cheese.	Umami	0.971
Manchego cheese	SAEQK	Sensory evaluation, MS identification	Identified as an umami peptide in Manchego cheese.	Umami	0.964
Manchego cheese	ENINEL	Sensory evaluation, MS identification	Identified as an umami peptide in Manchego cheese.	Umami	0.938
Manchego cheese	EQEEL	Sensory evaluation, MS identification	Identified as an umami peptide in Manchego cheese.	Umami	0.962
Manchego cheese	QEEL	Sensory evaluation, MS identification	Identified as an umami peptide in Manchego cheese.	Umami	0.94

Fermented food source	Confirmed umami or umami-enhancing peptides	Main validation evidence	Taste threshold or main taste effect	Class	Probability
Manchego cheese	NVVGET	Sensory evaluation, MS identification	Identified as an umami peptide in Manchego cheese.	Umami	0.95
Manchego cheese	VVGET	Sensory evaluation, MS identification	Identified as an umami peptide in Manchego cheese.	Umami	0.948
